# Salidroside attenuates neuroinflammation and improves functional recovery after spinal cord injury through microglia polarization regulation

**DOI:** 10.1111/jcmm.13368

**Published:** 2017-11-17

**Authors:** Chenggui Wang, Qingqing Wang, Yiting Lou, Jianxiang Xu, Zhenhua Feng, Yu Chen, Qian Tang, Gang Zheng, Zengjie Zhang, Yaosen Wu, Naifeng Tian, Yifei Zhou, Huazi Xu, Xiaolei Zhang

**Affiliations:** ^1^ Department of Orthopaedics The Second Affiliated Hospital and Yuying Children's Hospital of Wenzhou Medical University Wenzhou Zhejiang Province China; ^2^ Key Laboratory of Orthopaedics of Zhejiang Province Wenzhou China; ^3^ Chinese Orthopaedic Regenerative Medicine Society Hangzhou China

**Keywords:** salidroside, spinal cord injury, microglia, M1 polarization, M2 polarization, autophagic flux, AMPK

## Abstract

Spinal cord injury (SCI) is a severe neurological disease; however, few drugs have been proved to treat SCI effectively. Neuroinflammation is the major pathogenesis of SCI secondary injury and considered to be the therapeutic target of SCI. Salidroside (Sal) has been reported to exert anti‐inflammatory effects in airway, adipose and myocardial tissue; however, the role of Sal in SCI therapeutics has not been clarified. In this study, we showed that Sal could improve the functional recovery of spinal cord in rats as revealed by increased BBB locomotor rating scale, angle of incline, and decreased cavity of spinal cord injury and apoptosis of neurons *in vivo*. Immunofluorescence double staining of microglia marker and M1/M2 marker demonstrated that Sal could suppress M1 microglia polarization and activate M2 microglia polarization *in vivo*. To verify how Sal exerts its effects on microglia polarization and neuron protection, we performed the mechanism study *in vitro* in microglia cell line BV‐2 and neuron cell line PC12. The results showed that Sal prevents apoptosis of PC12 cells in coculture with LPS‐induced M1 BV‐2 microglia, also the inflammatory secretion phenotype of M1 BV‐2 microglia was suppressed by Sal, and further studies demonstrated that autophagic flux regulation through AMPK/mTOR pathway was involved in Sal regulated microglia polarization after SCI. Overall, our study illustrated that Sal could promote spinal cord injury functional recovery in rats, and the mechanism may relate to its microglia polarization modulation through AMPK‐/mTOR‐mediated autophagic flux stimulation.

## Introduction

Spinal cord injury (SCI) induces sudden sensorimotor and/or autonomic functional loss resulting in decreased quality of life and significant medical burdens to society 1. The pathogenesis of SCI comprises the following two stages: primary injury and secondary injury. Primary injury is mainly caused by direct injury to the spinal cord, whereas secondary injury is induced by several complex phenomena, such as haemorrhage and ischaemia, hypoxia, inflammation and oedema 2, 3, 4. Inflammation is an unavoidable process that may amplify the secondary injury of SCI, and neuroinflammation is primarily mediated by resident macrophages, which are called microglia in the central nervous system (CNS) 5. Macrophages can assume the following two phenotypes: a classically activated pro‐inflammatory (M1) phenotype and an alternatively activated anti‐inflammatory (M2) phenotype 6. Studies outside the CNS have shown that macrophages, which infiltrate tissues from the peripheral blood, participate in tissue regeneration 6, 7. Furthermore, studies also illustrate that microglia contribute to the repair of injury in the CNS, including SCI 5, 8.

Microglia in the injured spinal cord are mainly polarized to a M1 phenotype 9, 10, and polarized M1 assume an amoeboid shape, a change accompanied by the release of neurotoxic substances such as free radicals, acute‐phase proteins, inflammatory cytokines and chemokines 11. These inflammatory mediators can cooperate to cause neuronal damage 5. In contrast, microglia undergoing M2 polarization experience phenotypic changes enabling them to promote neuroregeneration, particularly axonal extension, after CNS injury 6, 12, 13. Within 7 days after SCI, transient activation of a small number of M2 microglia and long‐term activation of numerous M1 microglia are observed in the injured spinal cord 6. Treatment with either GM‐CSF or IL‐4, which may drive microglia M2 polarization, improves tissue sparing and locomotor recovery after SCI 14, 15, 16. Transferring M2 macrophages to the injured spinal cord has been shown to promote locomotor recovery in SCI rats 17. Therefore, promoting M2 microglia polarization may be a promising treatment strategy to facilitate functional recovery after SCI. However, few drugs have been discovered that promote M2 polarization of microglia 18, 19.

Studies have shown that herbs and herbal extracts used in traditional Chinese medicine are effective in the treatment of CNS diseases, such as Parkinson's disease 20, Alzheimer's disease 21 and SCI 22. Salidroside (Sal), the major phenylpropanoid glycoside extract from rhodiola, has various bioactive properties. Studies have shown that Sal may improve cognitive function 23, 24 and exert antiarrhythmic, anti‐inflammatory and neuroprotective effects 25, 27, 28; however, the ability of Sal to regulate microglia polarization and its neuroprotective effects in SCI remain unknown. This study aimed to investigate whether Sal can regulate microglia M1/M2 polarization and promote functional recovery of rats after SCI as well as the underlying mechanisms.

Autophagy, a catabolic process through which dysfunctional organelles and proteins are degraded to protect the cell against various stresses, has been reported to be involved in SCI recovery 29, 30, 31, 32. Recent studies have found that there is crosstalk between autophagy and macrophage polarization. Increased macrophage autophagy induced by rapamycin treatment decreases local M1 polarization and promotes locomotor function recovery 33. The inhibition of autophagy in the tumour microenvironment attenuates M2 macrophage polarization 34. Furthermore, evidence has shown that Cathepsin S‐mediated autophagic flux is an important mechanism for inducing M2 macrophage polarization 35, and impaired macrophage autophagy promotes pro‐inflammatory macrophage polarization in obese mice 36. Therefore, we focused our study on autophagy flux to explore the mechanism by which Sal regulates microglia polarization.

In this study, we found that Sal could promote functional recovery in SCI rats. In addition, we also showed that M2 polarization of microglia/macrophage was activated and M1 polarization of microglia/macrophage was suppressed by Sal treatment *in vivo*. *In vitro,* Sal‐induced M2 microglia polarization protected the PC12 neuronal cell line from apoptosis, and Sal also inhibited the M1 inflammatory secretion phenotype of microglia, the underlying mechanism of which may involve regulation of AMPK/mTOR pathway‐mediated autophagic flux. This study demonstrates that Sal may be a promising therapeutic drug to target microglia polarization in SCI.

## Materials and Methods

### Reagents and antibodies

Cell counting kit‐8 (CCK‐8) was obtained from Dojindo (Kumamoto, Japan). Compound c and rapamycin were purchased from Selleckchem (Houston, TX, USA), and LPS and chloroquine were purchased from Sigma‐Aldrich (St Louis, MO, USA). Monoclonal antibodies specific for LC3B, Bcl‐2 and Bax were procured from Cell Signaling Technologies (Beverly, MA, USA). Monoclonal antibodies specific for p‐AMPKα, AMPKα, p62, cleaved caspase 3, iNOS, COX‐2, CD68, Iba‐1, NEUN, NF‐200 and GFAP, and a polyclonal antibody specific for Arg‐1 were purchased from Abcam (Cambridge, UK). A polyclonal antibody specific for LAMP‐2 was obtained from Bioworld (Nanjing, China), and polyclonal antibodies specific for TNF‐α and IL‐6 were purchased from Santa Cruz Biotechnology (Dallas, TX, USA). AlexaFluor 568, AlexaFluor 488 donkey anti‐rabbit/mouse and horseradish peroxidase‐labelled secondary antibodies were purchased from Abcam. 4′, 6‐Diamidino‐2‐phenylindole (DAPI) was obtained from Beyotime (Shanghai, China). The PCR primers used herein were synthesized by TsingKe (Beijing, China), and all reagents used in the real‐time reverse transcription‐polymerase chain reaction (RT‐PCR) and cell culture experiments were purchased from Takara (Dalian, China) and Bio‐Rad (Hercules, California, USA), respectively. Sal (C14H20O7, CAS#: 10338–51–9, HPLC > 98%) was purchased from Nanjing Zelang Medical Technology (Nanjing, China). For the *in vitro* studies, compound c, rapamycin and Sal were dissolved in dimethyl sulphoxide (DMSO) at concentrations of 5 mM, 1 mM and 0.2 M, respectively, and were diluted appropriately with cell culture medium (final DMSO concentration ≤1‰). For the *in vivo* studies, Sal was dissolved in normal saline.

### Establishment of the SCI Model and drug treatment

Total 36 adult female S.D. rats (220–250 g) were ordered from the SLAC Laboratory Animal Company in Shanghai, China, and the animals were cared for and handled according to the guidelines set forth by the Chinese National Institutes of Health. All the rats were housed under controlled environmental conditions. To induce SCI, we anaesthetized all the animals with 8% (w/v) chloral hydrate (3.5 ml/kg, i.p.). Then, we performed a laminectomy at the T9 vertebrae after exposing the vertebral column. The spinal cord was fully exposed, and a moderate crush injury was induced over 1 min. with a vascular clip (30 g forces, Oscar, Shanghai, China). Sham group rats underwent the same surgical procedure but were not subjected to compression. Postoperatively, the animals’ urinary bladders were emptied twice daily. After surgery, the animals were immediately injected with Sal (25 mg/kg i.p.) and given daily 25 mg/kg doses until they were killed. The control group was injected with an equivalent dose of normal saline.

### Locomotion recovery assay

The extent of post‐SCI locomotion recovery experienced by the rats was investigated using the Basso, Beattie, and Bresnahan (BBB) locomotion rating scale 37 and the inclined plane test 38. The BBB tests, whose results were scored on a scale ranging from 0 to 21 points, were conducted 1, 3, 7, 14, 21 and 28 days after SCI. The inclined plane test was also performed to assess functional improvement in each rat at each of the above time‐points. The outcome measures were assessed by five independent examiners blinded to the experimental conditions.

### Histology and immunofluorescence

The rats (*n *=* *5 per group) were killed with 10% chloral hydrate (3.5 ml/kg, i.p.) at specific time‐points after SCI and then perfused with normal saline. For the H&E staining, Nissl staining and immunofluorescence analyses, tissue segments containing the lesion (1 cm on each side of the lesion), 0.5‐cm sections of the spinal cord were dissected out, post‐fixed with 4% paraformaldehyde for 6 hrs and then embedded in paraffin. Transverse sections (5 μm thick) were mounted on slides following staining. Histopathological examinations were performed by H&E and Nissl staining, according to the manufacturer's instructions. Bright‐field images were acquired using light microscopy (Olympus, Tokyo, Japan). For immunofluorescence analysis, the longitudinal sections (5 μm thick) were treated with the following primary antibodies: NF‐200 (1:2000) and GFAP (1:2000), and the transverse sections were treated with the following primary antibodies anti‐CD68 (1:200), anti‐Iba‐1 (1:200), anti‐Arg‐1 (1:200), anticleaved caspase 3 (1:200) and anti‐NeuN (1:100) before being washed four times with PBS and incubated with AlexaFluor 568 and AlexaFluor 488 donkey anti‐rabbit/mouse secondary antibodies for 1 hr at 37°C. Then, the sections were washed with PBS, incubated with DAPI for 1 min., rinsed with PBS and finally sealed with a coverslip. All images were captured on a confocal fluorescence microscope (Nikon, Japan).

### Cell culture

The immortalized murine BV‐2 microglial cell line was first generated by Blasi *et al*. and retains many of the morphological and functional characteristics of primary microglia 39. The PC12 cell line was purchased from the Shanghai Institute of Cell Biology. The BV‐2 cells were cultivated in EMEM, and the PC12 cells were cultured in 1640 medium (Invitrogen, Carlsbad, CA, USA) supplemented with 10% foetal bovine serum (FBS, Gibco BRL Co., Ltd., USA), 100 units/ml penicillin and 100 μg/ml streptomycin; incubated at 37°C in a humidified atmosphere containing 5% CO_2_; and passaged twice a week.

### BV‐2 cell treatment

To establish the BV‐2 inflammation model, we added LPS (1 μg/ml) to the BV‐2 culture medium for 24 hrs. We pre‐treated the cells with different dose of Sal (2, 20 and 200 μM) for 24 hrs before treating them with LPS (1 μg/ml) to investigate the effect of the drug on cellular inflammation. To study the effects of autophagy on BV‐2 cells, we pre‐treated the cells with 50 μM chloroquine (CQ, an autophagy inhibitor) and 5 μm of compound C (an AMP‐activated protein kinase inhibitor) for 2 hrs before treating them with Sal and LPS. All experiments were performed in triplicate.

### Microglia/neuron coculture

To investigate the relationship between microglia and neurons, we utilized a transwell coculture system (Corning, 0.4‐μm pores, USA). BV‐2 and PC12 cells were separately seeded into 24‐well (1 × 10^5^ BV‐2 cells/insert and 2 × 10^5^ PC12 cells/well) transwell plates for the coculture experiment. The BV‐2 cells were pre‐treated with or without 2, 20 and 200 μM Sal for 24 hrs or 50 μM CQ for 2 hrs before being stimulated with 1 μg/ml LPS for another 24 hrs. Then, the BV‐2 cells were treated with or without LPS for 1, 3, 6, 12 and 24 hrs. After stimulation, the BV‐2 cell inserts were rinsed with PBS to eliminate the effects of any remaining LPS, Sal or CQ before being placed into the PC12 wells. After 24 hrs of coculture, we measured neuronal apoptosis by CCK‐8, immunofluorescence and Western blot assays.

### Cell viability measurements

Cell viability was assessed by CCK‐8 assay, according to the manufacturer's protocol. Briefly, BV‐2 cells were seeded in 96‐well plates (5000 cells/well) and incubated for 24 hrs. Then, different doses of Sal (0, 0.2, 2, 20, 200 and 2000 μM) were added to the wells for the cell viability assay. For the coculture system, BV‐2 cells were pre‐treated with 2000 μM Sal, followed by LPS, before being placed in wells containing PC12 cells. After treatment, 10 μl of tetrazolium substrate was added to the wells, and the plates were cultured for 1 hr. The absorbance was measured at 450 nm using a microplate reader (Thermo Scientific, Multiskan Go, Waltham, MA, USA).

### Assessment of MMP

MMP (ΔΨm) was measured using rhodamine 123, a cationic lipophilic fluorochrome that can be absorbed by mitochondria. After incubating for 30 min. in the dark, the cells were washed twice, and fluorescence images were immediately recorded and quantified under an inverted fluorescence microscope (Nikon).

### Calcein AM/propidium iodide (PI) cellular viability assay

Cell viability was assessed by double‐staining assay using calcium fluorescein‐AM/PI. PC12 cells were seeded in a 24‐well plate and allowed to attach for 24 hrs. Then, BV‐2 cells were pre‐treated with or without 200 μM Sal for 24 hrs before being stimulated with LPS and placed in the wells containing the PC12 cells for 24 hrs. Then, the PC12 cells washed gently with PBS two times, after which 2 μM calcein AM and 15 μg·M^−1^ PI were added to the wells, and the culture plates were incubated at 37°C for 30 min. Finally, the dye solution was removed, and the samples were washed with PBS three times. A fluorescence microscope (Nikon) was used to assess the slides.

### Cellular immunofluorescence

BV cells were seeded on slides in a six‐well plate and allowed to attach for 24 hrs. The cells were subsequently treated with or without 200 μM Sal for 24 hrs before being stimulated with LPS for 24 hrs. For immunofluorescence analysis, the samples were fixed with 4% paraformaldehyde and permeabilized with 0.5% Triton X‐100 for 10 min. After being blocked with 10% goat serum albumin for 60 min. at 37°C, the slides were incubated with primary antibodies against CD68 (1:300), p62 (1:200), LC3B (1:200) and Arg‐1 (1:300) overnight at 4°C. The following day, the slices were washed thrice with PBS and then incubated with AlexaFluor 568 and AlexaFluor 488 donkey anti‐rabbit/mouse secondary antibodies for 1 hr at 37°C before being labelled with DAPI for 1 min. at room temperature. Finally, three fields of view per slide were randomly selected for observation under a fluorescence microscope (Olympus Inc., Tokyo, Japan), and staining intensities were measured by observers blinded to the experimental groups using Image‐Pro Plus 6.0 (Media Cybernetics, Rockville, MD, USA).

### Tunel method

The TUNEL method is useful for measuring apoptotic DNA fragmentation. PC12 cells were seeded on slides in a 24‐well plate and allowed to attach for 24 hrs. Then, BV‐2 cells were pre‐treated with or without 200 μM Sal for 24 hrs before being treated with LPS and placed in the wells containing the PC12 cells for 24 hrs. Then, the PC12 cells were washed gently with PBS two times and fixed with freshly prepared 4% paraformaldehyde for 30 min. before being incubated with 0.1% Triton X‐100 for 5 min. The cells were washed with PBS three times at every step. The cells were then stained using a DeadEnd^TM^ Fluorometric TUNEL System (Promega, Madison, WI, USA) and DAPI, according to the manufacturer's instructions. Apoptosis was measured using a fluorescence microscope (DM 2500; Leica, Wetzlar, Germany).

### Real‐time RT‐PCR analysis

Total RNA extracts were obtained using TRIzol reagent (Invitrogen), and 1 μg of total RNA was used to synthesize cDNA (TaKaRa, Osaka, Japan). qPCR was performed using SYBR Green (Bio‐Rad, Hercules, USA). The cDNA samples were amplified on an Applied CFX96^®^ real‐time PCR system (Bio‐Rad) under the following conditions: 95°C for 3 min., followed by 40 cycles of 95°C for 15 sec. and 60°C for 45 sec. The relative expression levels of the target genes were normalized to those of the housekeeping gene GAPDH, and the target genes from the experimental group were compared with the corresponding target genes from the control group using the 2^−ΔΔCT^ method. The primers for iNOS, TNF‐α, COX‐2, Arg‐1 and GAPDH are listed in Table 1.

**Table 1:** Primers used for real‐time PCR analysis

Genes	Forward primers	Reverse primers
iNOS	CCCTTCAATGGTTGGTACATGG	ACATTGATCTCCGTGACAGCC
TNF‐α	CTCAAGCCCTGGTATGAGCC	GGCTGGGTAGAGAACGGATG
COX‐2	AACCCAGGGGATCGAGTGT	CGCAGCTCAGTGTTTGGGAT
Arg‐1	CAGAAGAATGGAAGAGTCAG	CAGATATGCAGGGAGTCACC
GAPDH	ATGGTGAAGGTCGGTGTGA	CTCCACTTTGCCACTGCAA

### Western blot analysis

The tissue and cell supernatants were collected for protein assay. The extracts were first quantified with BCA reagents. Then, cellular samples containing 30 μg of protein and tissue samples containing 80 μg of protein were separated on SDS‐PAGE before being transferred to PVDF membranes and incubated with the appropriate primary antibodies overnight, after which they were incubated with horseradish peroxidase‐conjugated secondary antibodies for 2 hrs. The bands were detected by electrochemiluminescence reagent (Invitrogen), and the signals were visualized by a Chemi DocXRS^+^ Imaging System (Bio‐Rad).

### Statistical analysis

Numerical data from at least three individual experiments are shown as the mean ± S.D. and were analysed by one‐way analysis of variance (anova) followed by Tukey's post hoc analysis. Between‐group differences in BBB scores and inclined plane test results were analysed using generalized linear mixed models. Statistical significance was established at *P <* *0.05, **P <* *0.01 *versus* the indicated group.

## Results

### Sal promotes locomotor function recovery in SCI rats

**Figure 1:**
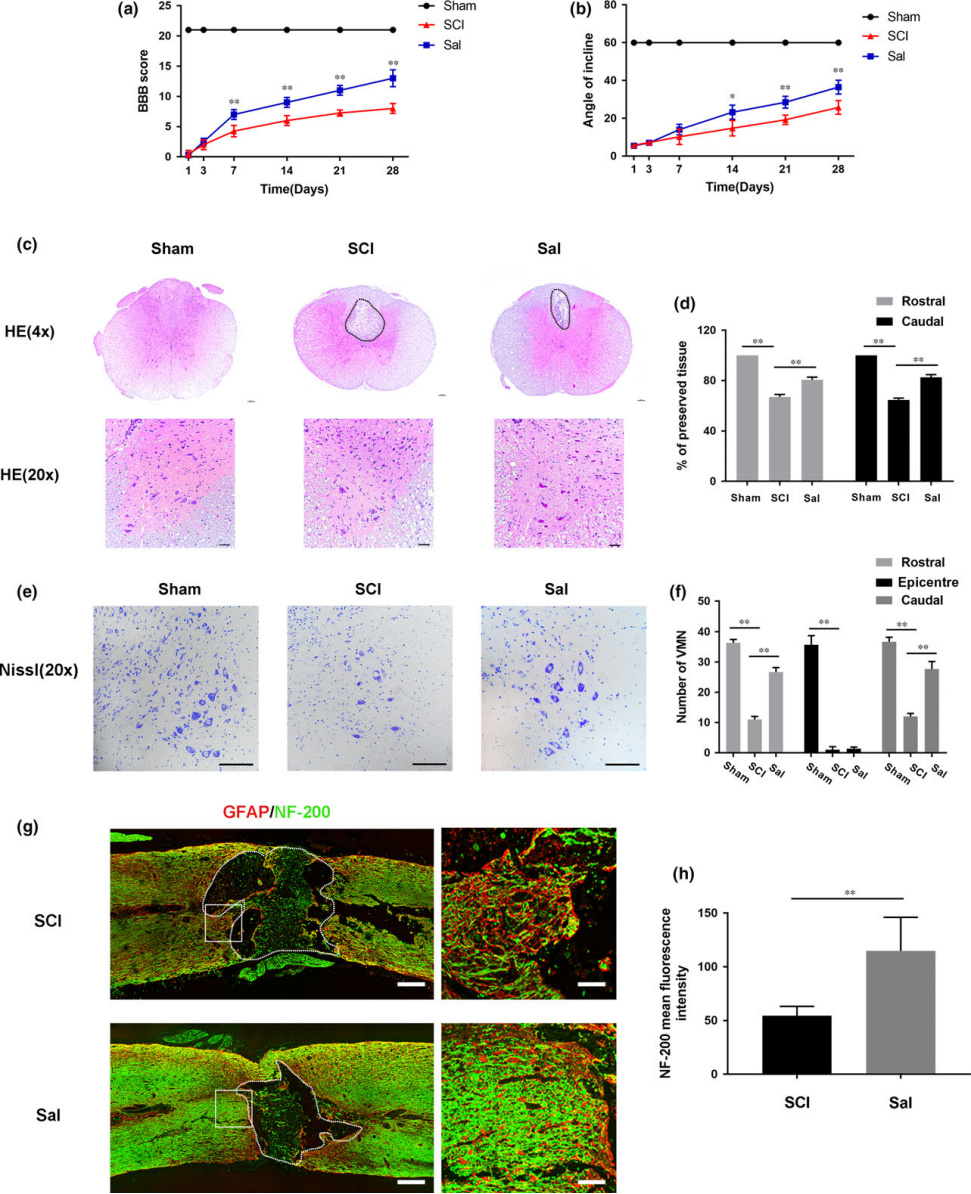
Sal attenuates tissue structural damage, neuron loss and improves functional recovery after experimental acute traumatic SCI (**A**) Basso, Beattie and Bresnahan (BBB) scores. (**B**) Inclined plane test scores. (**C**) H&E stains at 7 days. Scale bars are 100 μm (4×) and 50 μm (20×). Dashed lines area showed cavity of spinal cord. (**D**) Graphic presentation of the percentage of preserved tissue relative to the transverse area of the spinal cord on the seventh postoperative day. (**E**) Nissl staining to assess the loss of neurons at 7 days. Scale bars are 50 μm. (**F**) Counting analysis of VMN at rostral 5 mm, caudal 5 mm and lesion site. (**G**) Representative images containing neurofilament (NF‐200, green) and GFAP (red) immunofluorescence on spinal cord sections. Dashed lines area showed cavity of spinal cord. Scale bar are = 1000 μm and 50 μm. (**H**) Quantitative analysis of NF‐200 staining intensity. Data are presented as the mean ± S.D., *n *=* *3 independent experiments. Significant differences between groups are indicated as *P <* *0.05 and **P <* *0.01.

As locomotor functional recovery was associated with neuronal regeneration, we evaluated behavioural changes after injury using BBB scores and the inclined plane test. We found that all the rats lost function in their hind legs immediately after SCI and displayed time‐dependent neuronal functional restoration in response to Sal treatment. The results of the experiments showed that the experimental groups exhibited significant differences from one another with respect to their degree of motor functional recovery (Fig. 1A and B). Our BBB score and angle of incline estimates showed that the Sal treatment group experienced significantly greater functional recovery than other groups, indicating that Sal can improve motor functional recovery after SCI. We evaluated the histological morphological changes experienced by each group using HE and Nissl staining. As shown in Figure 1C and D, in contrast to the rats in the sham group, the rats in the SCI group exhibited severely damaged central grey matter and dorsal white matter. Compared with the SCI group, the Sal treatment group exhibited attenuated central grey matter and dorsal white matter cavitation, indicating that Sal exerted therapeutic effects on SCI. The numbers of ventral motor neurons (VMNs) were counted. As shown in Figure 1E and F, SCI induced significant VMN loss; however, Sal significantly attenuated the VMN loss induced by SCI, demonstrating that Sal can reduce neuron loss and ameliorate the pathological morphological changes characteristic of tissue injury. Double staining for GFAP (red) and NF‐200 (green) was performed to assess the regeneration of neurofilaments (Fig. 1G and H). The results showed that in the SCI group, the neurofilaments were completely lost in the injury epicentre; however, the Sal‐treated rats presented a pronounced increased number of NF‐200‐labelled fibres around the lesion site. Collectively, these findings indicate that Sal facilitates motor functional recovery by promoting neuron rehabilitation.

### Sal attenuates neuronal apoptosis and increases the M2/M1 polarization ratio in SCI rats

**Figure 2:**
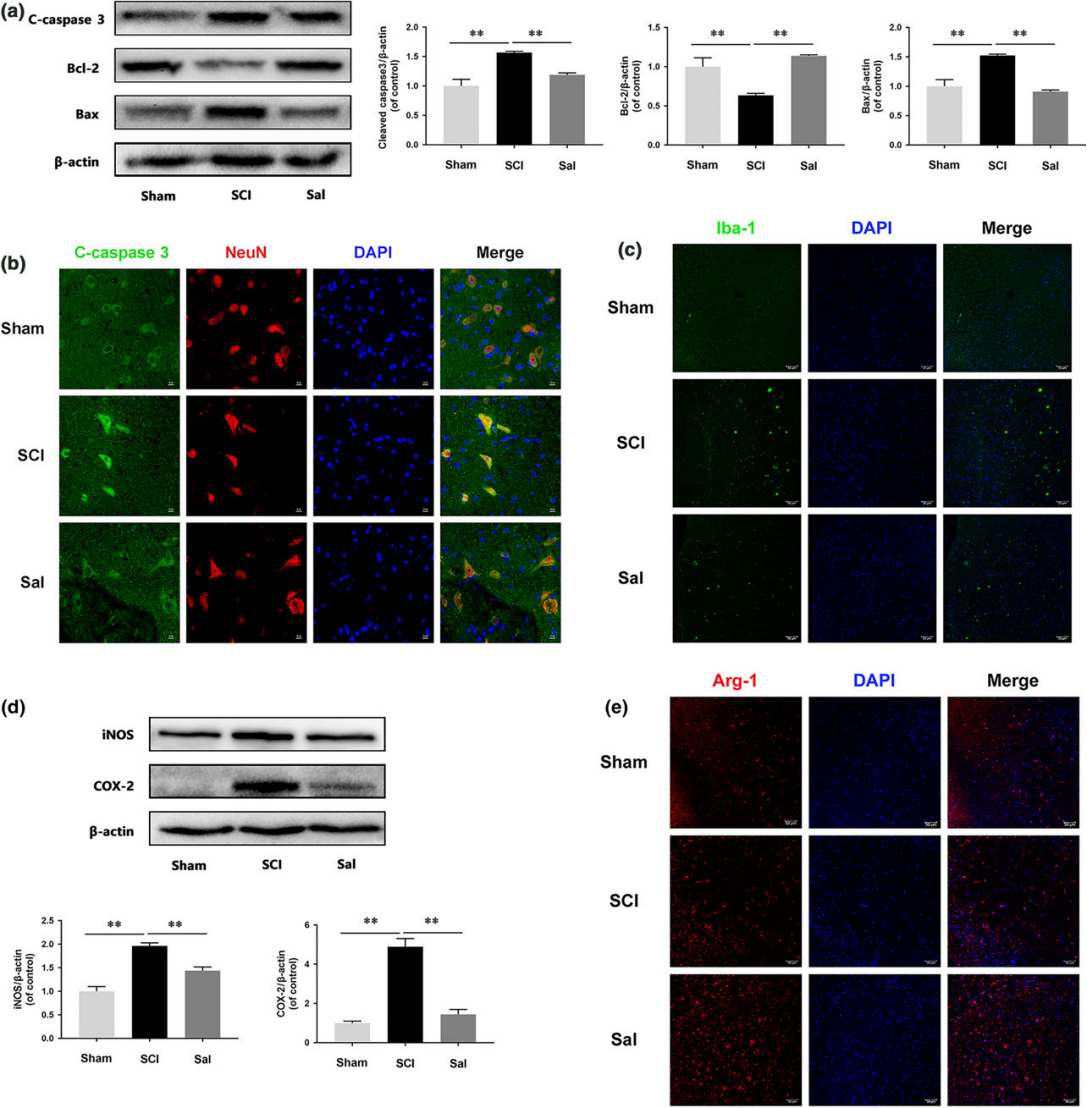
Sal prevents neuronal apoptosis and regulates microglia/macrophage polarization (**A**) Western blot analysis of cleaved caspase 3, Bcl‐2 and Bax expression in each group of rats. Sal evidently prevented SCI‐induced neuronal apoptosis. (**B**) Double‐staining for cleaved caspase 3 (green)/NeuN (red) in sections of injured spinal cord tissue from each group of rats (scale bar: 10 μm). (**C**) Immunofluorescence staining for Iba‐1 in injured spinal cord tissue from each group rats (scale bar: 50 μm). Sal significantly reduced the numbers of M1 microglia after SCI. (**D**) Representative Western blots of and quantitative data for iNOS, COX‐2 and β‐actin expression in each group of rats. Inflammatory mediator release was suppressed by Sal treatment. (**E**) Immunofluorescence staining for Arg‐1 in sections of injured spinal cord tissue from each group rats (scale bar: 50 μm). Sal significantly increased M2 cell numbers after SCI. Densitometric analysis of all Western blot bands, whose densities were normalized to those of β‐actin. Data are presented as the mean ± S.D., *n *=* *3 independent experiments. Significant differences between groups are indicated as *P <* *0.05 and **P <* *0.01.

To determine whether Sal prevents neuronal apoptosis, we analysed Bcl‐2, Bax and cleaved caspase 3 levels by Western blotting (Fig. 2A). The results indicated that Sal significantly reversed the apoptosis induced by mitochondria dysfunction after SCI. We also performed double‐immunofluorescence staining for cleaved caspase 3 and NeuN. As shown in Figure 2B, SCI increased neuronal apoptosis, as demonstrated by the results of our double‐staining experiments, which showed that neurons in the SCI group exhibited increased staining for cleaved caspase 3 and NeuN compared with neurons in the control group. To investigate the neuroprotective effects of Sal, we detected the expression of CD68, a M1 microglia/macrophage marker, by immunofluorescence analysis (Fig. S1), the results of which showed that increased numbers of M1 microglia/macrophage were present at lesion sites in acute SCI and that Sal treatment decreased the numbers of M1 microglia/macrophage present at these sites. Furthermore, a M1 microglia marker, Iba‐1, was decreased by Sal treatment compared with SCI groups (Fig. 2C). Consistent with our Western blotting results (Fig. 2D), the results of our studies involving additional markers showed that iNOS and COX‐2 expression levels were increased after SCI and decreased by Sal treatment. We also performed immunofluorescence for Arg‐1, a M2 microglia/macrophage marker. Interestingly, the numbers of M2 microglia/macrophage were increased after Sal treatment compared with before treatment (Fig. 2E). These data indicated that Sal attenuates neuronal apoptosis may through suppressing M1 microglia/macrophage polarization and increasing M2 microglia/macrophage polarization.

### LPS induces M1 microglia polarization and promotes pro‐inflammatory mediator release

**Figure 3:**
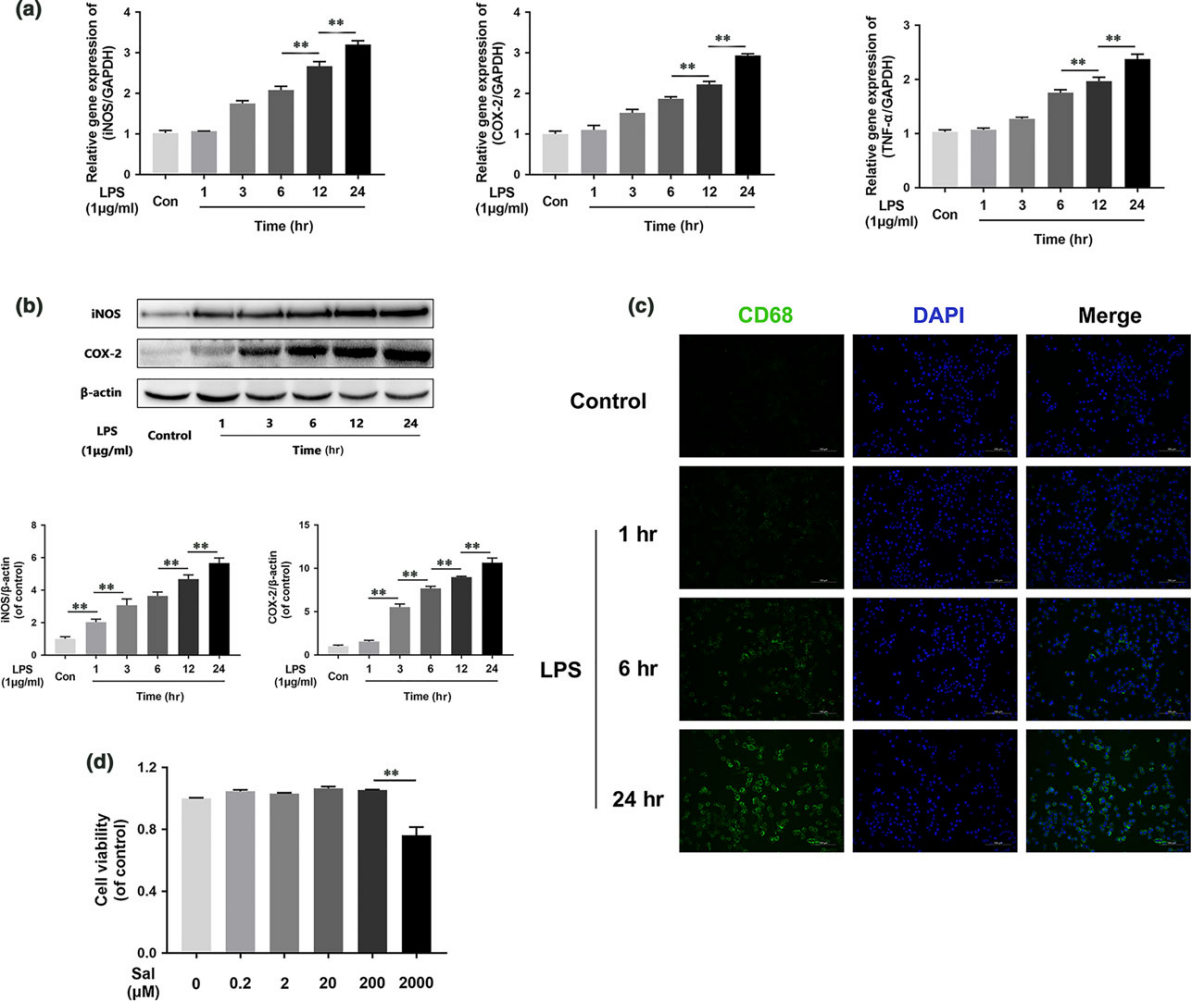
LPS induces M1 microglia polarization and pro‐inflammatory mediator release. (**A**) iNOS, COX‐2 and TNF‐α gene expression levels in BV‐2 cells. Gene expression levels were assessed *via* qRT‐PCR and normalized to GAPDH gene expression levels. Related gene expression levels were up‐regulated by LPS stimulation in a time‐dependent manner. (**B**) Representative Western blots of and quantitative data for iNOS, COX‐2 and β‐actin expression in each group of microglia. iNOS and COX‐2 protein expression levels were enhanced by LPS stimulation. (**C**) Immunofluorescence staining for CD68 in each of group microglia (scale bar: 100 μm). LPS induced M1 microglia polarization in a time‐dependent manner. Densitometric analysis of all Western blot bands, whose densities were normalized to those of β‐actin. Data are presented as the mean ± S.D., *n *=* *3 independent experiments. Significant differences between groups are indicated as *P <* *0.05 and **P <* *0.01.

As shown in Figure 3A, the mRNA expression levels of pro‐inflammatory mediators, such as TNF‐α, iNOS and COX‐2, increased in a time‐dependent manner beginning at 1 hr after LPS stimulation and ultimately peaked at 24 hrs after SCI in the corresponding group compared with control group. Moreover, iNOS and COX‐2 protein expression levels increased in a time‐dependent manner after LPS stimulation (Fig. 3B), results consistent with those of the immunofluorescence studies of the expression of CD68 (Fig. 3C). To evaluate Sal cytotoxicity, we treated microglia with or without 0.2, 2, 20, 200 and 2000 μM Sal for 48 hrs and then evaluated the outcome of the treatment by CCK‐8 assay (Fig. 3D), whose results indicated that Sal did not cause significant cytotoxicity at concentrations below 200 μM. Those findings suggested that LPS‐induced microglia‐mediated pro‐inflammatory mediator release may be linked to pro‐inflammatory microglia polarization.

### M1 microglia trigger mitochondrial dysfunction in neurons in coculture

**Figure 4:**
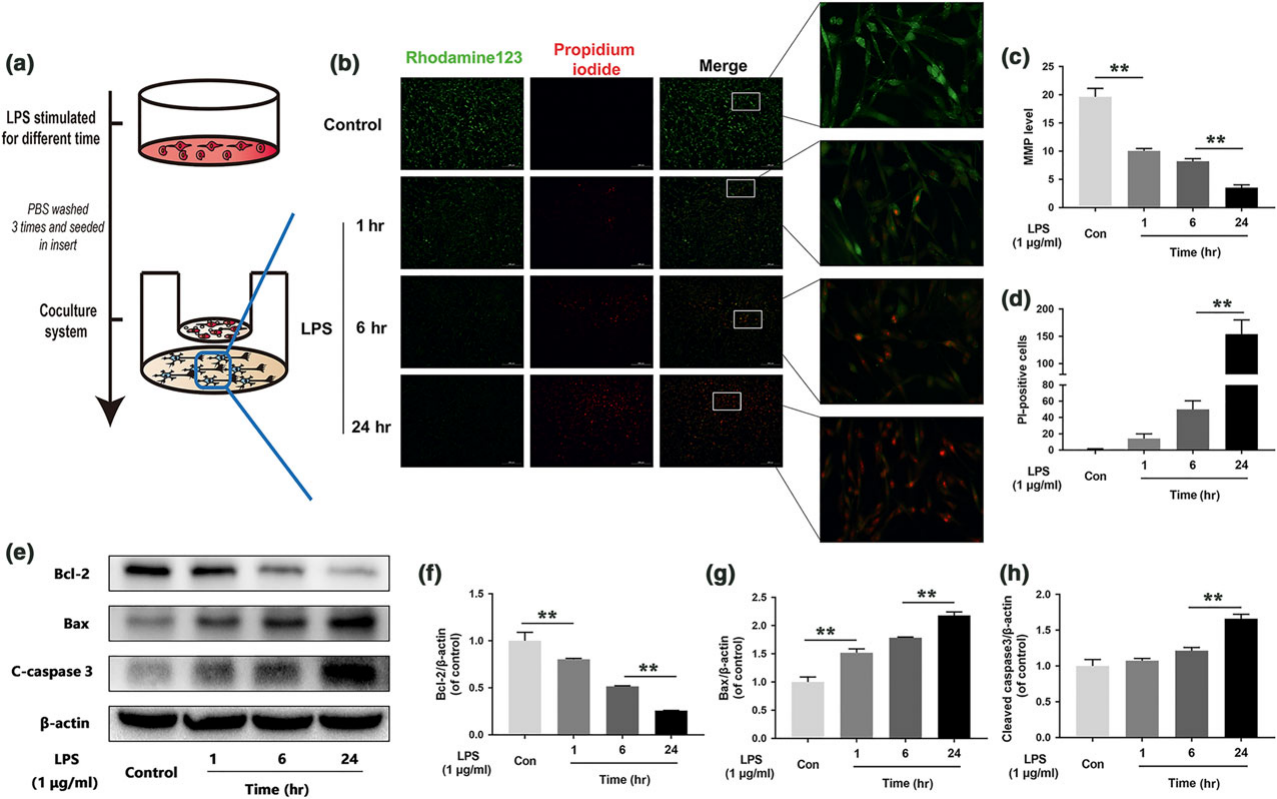
M1 microglia polarization induces neuronal apoptosis. (**A**) Schematic of BV‐2 cell treatments and the coculture system. BV‐2 cells were stimulated by LPS for 24 hrs, then washed three times and cocultured with PC12 cells. (**B, C, D**) Fluorescence staining results pertaining to MMP, as demonstrated by rhodamine 123 and PI. Representative images of stained neurons from the indicated groups (scale bar: 200 μm). MMP decreased significantly, and neuronal death increased in cells cocultured with M1 microglia. (**E, F, G, H**) Representative Western blots of and quantitative data for Bcl‐2, Bax, cleaved caspase 3 and β‐actin expression in each group of neurons. The neuronal apoptosis induced by mitochondria dysfunction was significantly increased in cells cocultured with M1 microglia. Densitometric analysis of all Western blot bands, whose densities were normalized to those of β‐actin. Data are presented as the mean ± S.D. and, *n *=* *3 independent experiments. Significant differences between groups are indicated as *P <* *0.05 and **P <* *0.01.

To evaluate the effects of M1 microglia on neurons, we used a coculture system. We treated the microglia as described in Figure 4A. We used rhodamine 123, a lipophilic cationic probe, to assess MMP. As shown in Figure 4B–D, we cocultured M1 microglia with neurons and found that M1 microglia leading to MMP dissipation in neuron, findings supported by our observation of time‐dependent decreases in green fluorescence intensity. The Bax/Bcl‐2 ratio and cleaved caspase 3 protein expression levels were positively correlated with the amount of time for which the microglia were treated with LPS (Fig. 4E–H). These data indicated that M1 microglia‐induced neuronal apoptosis was caused by mitochondria dysfunction.

### Sal prevents neuron apoptosis induced by M1 microglia

**Figure 5:**
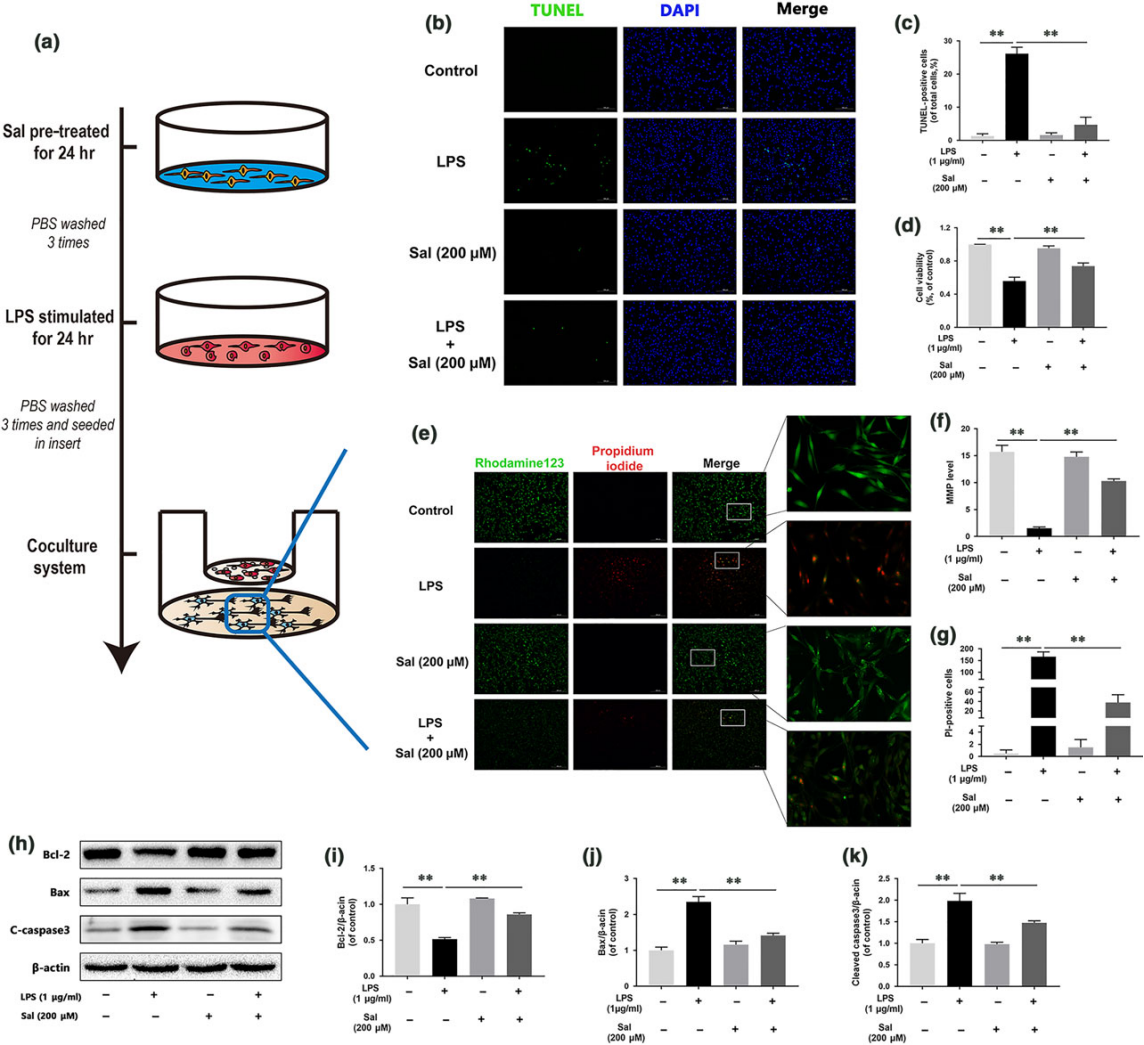
Sal prevents M1 microglia‐induced neuronal apoptosis (**A**) Schematic of BV‐2 cells treatments and the coculture system. BV‐2 cells were pre‐treated with Sal for 24 hrs, followed by washing three times and stimulation with LPS for 24 hrs, and then cocultured with PC12 cells. (**B, C**) TUNEL assay was performed in neurons cocultured with microglia (scale bar: 100 μm). Sal ameliorates neuronal apoptosis induced by M1 microglia. (**D**) CCK‐8 results pertaining to neurons cocultured with microglia. Cell viability was evidently increased in neurons cocultured with Sal‐pre‐treated microglia. (**E, F, G**) Fluorescence staining results for MMP, as demonstrated by rhodamine 123 and PI. Representative images of stained neurons from the indicated groups (scale bar: 200 μm). MMP increased significantly, and neuronal apoptosis decreased in cells cocultured with Sal‐pre‐treated microglia. (**H, I, J, K**) Representative Western blots of and quantitative data for Bcl‐2, Bax, cleaved caspase 3 and β‐actin expression in each group of neurons. The neuronal apoptosis induced by mitochondrial dysfunction was significantly attenuated in cells cocultured with Sal‐pre‐treated microglia. Densitometric analysis of all Western blot bands, whose densities were normalized to those of β‐actin. Data are presented as the mean ± S.D., *n *=* *3 independent experiments. Significant differences between groups are indicated as *P <* *0.05 and **P <* *0.01.

To investigate the effects of Sal on neuronal apoptosis induced by M1 microglia, we treated the cells as shown in Figure 5A. Then, we double‐stained the neurons with calcein AM/PI (Fig. S2). The results showed that PC12 cells cocultured with M1 microglia underwent apoptosis at a significantly increased rate compared with cells in the control group. Moreover, cells that were cocultured with Sal‐pre‐treated microglia underwent apoptosis at a significantly decreased rate compared with cells in the control group. Furthermore, the TUNEL assay (Fig. 5B and C) results showed that the incidence of apoptosis was significantly increased in neurons cocultured with M1 microglia and decreased in neurons cocultured with Sal‐pre‐treated microglia compared with control neurons. In contrast, cell viability was increased in cells cocultured with Sal‐pre‐treated microglia compared with control cells (Fig. 5D), suggesting that Sal protects against M1 microglia‐induced neuronal apoptosis. Moreover, the degree of MMP dissipation induced by M1 microglia was attenuated by Sal pre‐treatment (Fig. 5E–G). In addition, Western blotting was performed to detect apoptosis‐related protein expression in neurons. As shown in Figure 5H–K, the Bax/Bcl‐2 ratio and cleaved caspase 3 expression levels were significantly restored in neurons cocultured with Sal‐pre‐treated microglia. These data indicated that Sal‐pre‐treated microglia can prevent mitochondria dysfunction‐induced neuronal apoptosis.

### Sal inhibits LPS‐induced microglia polarization and promotes M2 microglia polarization

**Figure 6:**
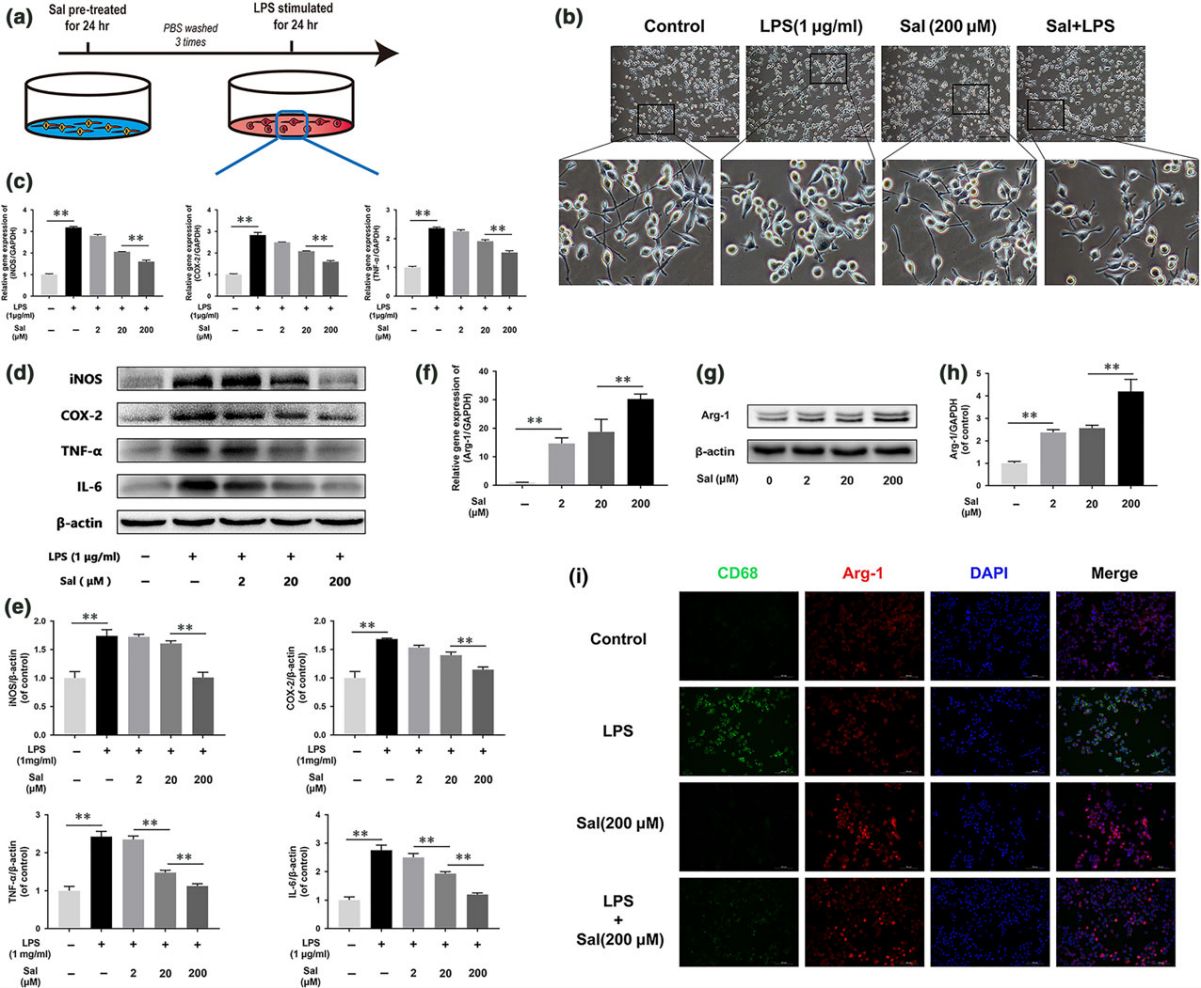
Sal regulates microglia polarization (**A**) Schematic of BV‐2 cell treatments. BV‐2 cells were pre‐treated with Sal for 24 hrs, followed by washing three times and stimulation with LPS for 24 hrs. (**B**) Morphological results for microglia in the Sal‐ and/or LPS‐treated BM‐EPC group (scale bar: 100 μm). Microglial treated with LPS displayed retracted branches, while microglia pre‐treated with Sal displayed extended branches. (**C**) iNOS, COX‐2 and TNF‐α gene expression levels in BV‐2 cells. Gene expression was assessed *via* qRT‐PCR and normalized to GAPDH expression. The expression levels of the related genes were up‐regulated by LPS stimulation and down‐regulated by Sal pre‐treatment. (**D, E**) Representative Western blots of and quantitative data for iNOS, COX‐2, TNF‐α, IL‐6 and β‐actin expression in each group of microglia. Sal significantly suppressed pro‐inflammatory mediator release and prevented M1 microglia polarization. (**F**) Arg‐1 gene expression levels in BV‐2 cells. Sal enhanced M2 polarization‐related gene expression levels in a dose‐dependent manner. (**G, H**) Representative Western blots of and quantitative data for Arg‐1 and β‐actin expression in BV‐2 cells. Sal increased M2 polarization marker protein expression levels in a dose‐dependent manner. (**G**) Double‐staining for CD68 (green)/Arg‐1 (red) in microglia from each group (scale bar: 100 μm). Sal pre‐treatment evidently decreased M1 polarization and increased M2 polarization. Densitometric analysis of all Western blot bands, whose densities were normalized to those of β‐actin. Data are presented as the mean ± S.D., *n *=* *3 independent experiments. Significant differences between groups are indicated as *P <* *0.05 and **P <* *0.01.

The effects of Sal on microglia polarization were studied *in vitro* using mainly BV‐2 microglial cells, as shown in Figure 6A. The morphological study results (Fig. 6B) showed that BV‐2 cells underwent M1 polarization and assumed an amoeboid shape after 24 hrs of LPS stimulation, changes that were prevented by Sal pre‐treatment. Moreover, the results indicated that unlike BV‐2 microglial cells stimulated by LPS (1 μg/ml), BV‐2 cells pre‐treated with 2–200 μM Sal displayed significantly suppressed TNF‐α, iNOS and COX‐2 mRNA expression (Fig. 6C). Cytokines such as iNOS, COX‐2, TNF‐α and IL‐6 are indicators of M1 microglia polarization. Similar to the results of the Western blot assay (Fig. 6D and E), the results of this experiment showed that TNF‐α, iNOS, COX‐2 and IL‐6 protein expression was significantly inhibited by Sal pre‐treatment in the indicated group compared with the control group. Furthermore, we measured M2 microglia polarization levels using qPCR (Fig. 6F) and Western blotting (Fig. 6G and H), the results of which indicated that Sal significantly increased M2 microglia‐related marker expression levels, as well as Arg‐1 expression levels, in the indicated group compared with the control group. Moreover, we detected the M2/M1 microglia ratio by performing double‐staining experiments involving CD68 and Arg‐1 (Fig. 6I). The results showed that Sal increased the M2/M1 microglia ratio in the indicated group compared with the control group. Taken together, these results showed that Sal suppressed M1 microglia polarization and regulated the M2/M1 ratio.

### Autophagic flux is involved in Sal‐induced microglial polarization

**Figure 7:**
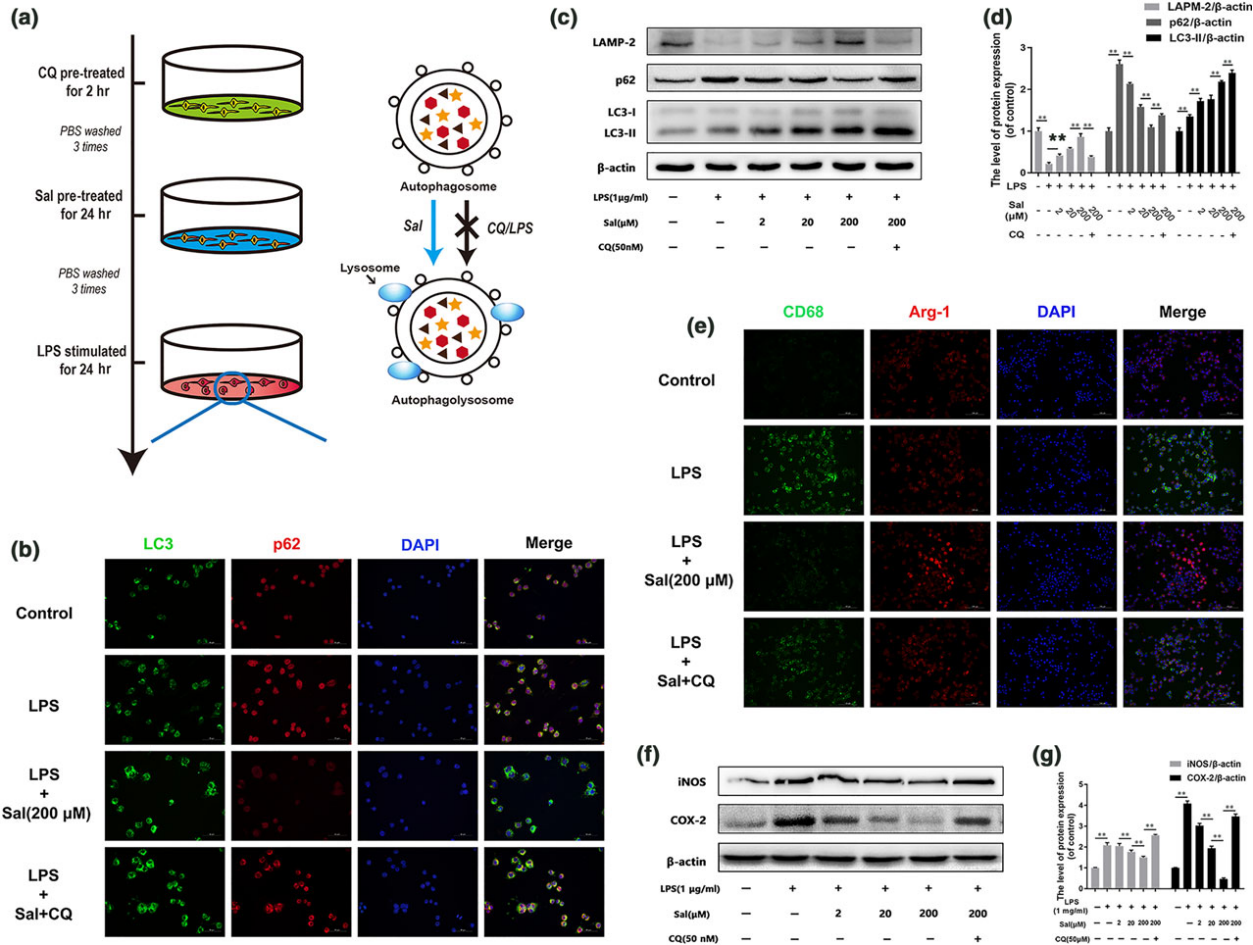
The microglia polarization‐related effects of Sal are related to autophagic flux (**A**) Schematic of BV‐2 cell treatments and the relationship between autophagic flux and Sal pre‐treatment. BV‐2 cells were pre‐treated with CQ for 2 hrs, followed by washing three times and treatment with Sal for 24 hrs. (**B**) Immunofluorescence staining for LC3 and p62 in each group of microglia (scale bar: 50 μm). Autophagic flux was blocked by LPS stimulation and activated by Sal pre‐treatment. (**C, D**) Representative Western blots of and quantitative data for LAMP‐2, p62, LC3 and β‐actin expression in BV‐2 cells. Lysosomal function was damaged by LPS stimulation and restored by Sal pre‐treatment. (**E**) Double‐staining for CD68 (green)/Arg‐1 (red) in microglia from each group (scale bar: 100 μm). Sal pre‐treatment evidently decreased M1 polarization and increased M2 polarization through the autophagy‐lysosome pathway. (**F, G**) Western blot analysis of iNOS, COX‐2 and β‐actin expression in each group of microglia. Sal evidently prevents M1 polarization and pro‐inflammatory mediator release, effects that were reversed by CQ. Densitometric analysis of all Western blot bands, whose densities were normalized to those of β‐actin. Data are presented as the mean ± S.D., *n *=* *3 independent experiments. Significant differences between groups are indicated as *P <* *0.05 and **P <* *0.01.

A previous study showed that impaired macrophage autophagy promotes M1 polarization 36. To investigate the relationship between autophagic flux and microglia polarization, we detected the expression levels of the autophagic flux‐related proteins LAMP‐2, p62 and LC3 *in vivo* (Fig. S3A–D). The Western bolting results showed that LAMP‐2 expression levels were decreased in the SCI group compared with the control group. Interestingly, p62 and LC3 expression levels were increased in the SCI group compared with control group, suggesting that autophagic flux may be inhibited in acute SCI. Moreover, LAMP‐2 and LC3‐II expression levels were increased, and p62 expression levels were decreased in the Sal‐treated group compared with the SCI group. These data indicated that the autophagic degradation pathway was inhibited by acute SCI and restored by Sal treatment. To determine the relationship between autophagic flux and microglial polarization, we used CQ, a specific lysosome inhibitor, to block autophagic flux, as shown in Figure 7A. The double‐immunofluorescence staining results for LC3 and p62 expression showed that Sal increased the number of LC3 puncta and deceased p62 fluorescence intensity in the indicated group compared with the control group (Fig. 7B). Moreover, Western blot assay showed that autophagic flux can be activated by Sal pre‐treatment, an effect that can be inhibited by CQ (Fig. 7C and D). Furthermore, we performed double‐staining for CD68 and Arg‐1 (Fig. 7E), the results of which indicated that blocking autophagic flux may enhance M1 polarization and reduce M2 polarization. The results of our Western blot assay for iNOS and COX‐2 expression indicated that Sal suppressed M1 microglia polarization in the indicated group compared with the control group, an effect that was significantly inhibited by CQ (Fig. 7F and G). These data suggested that microglia polarization may be facilitated by the autophagy‐lysosome pathway.

### Sal activates the AMPK/mTOR pathway *in vivo* and *in vitro*

**Figure 8:**
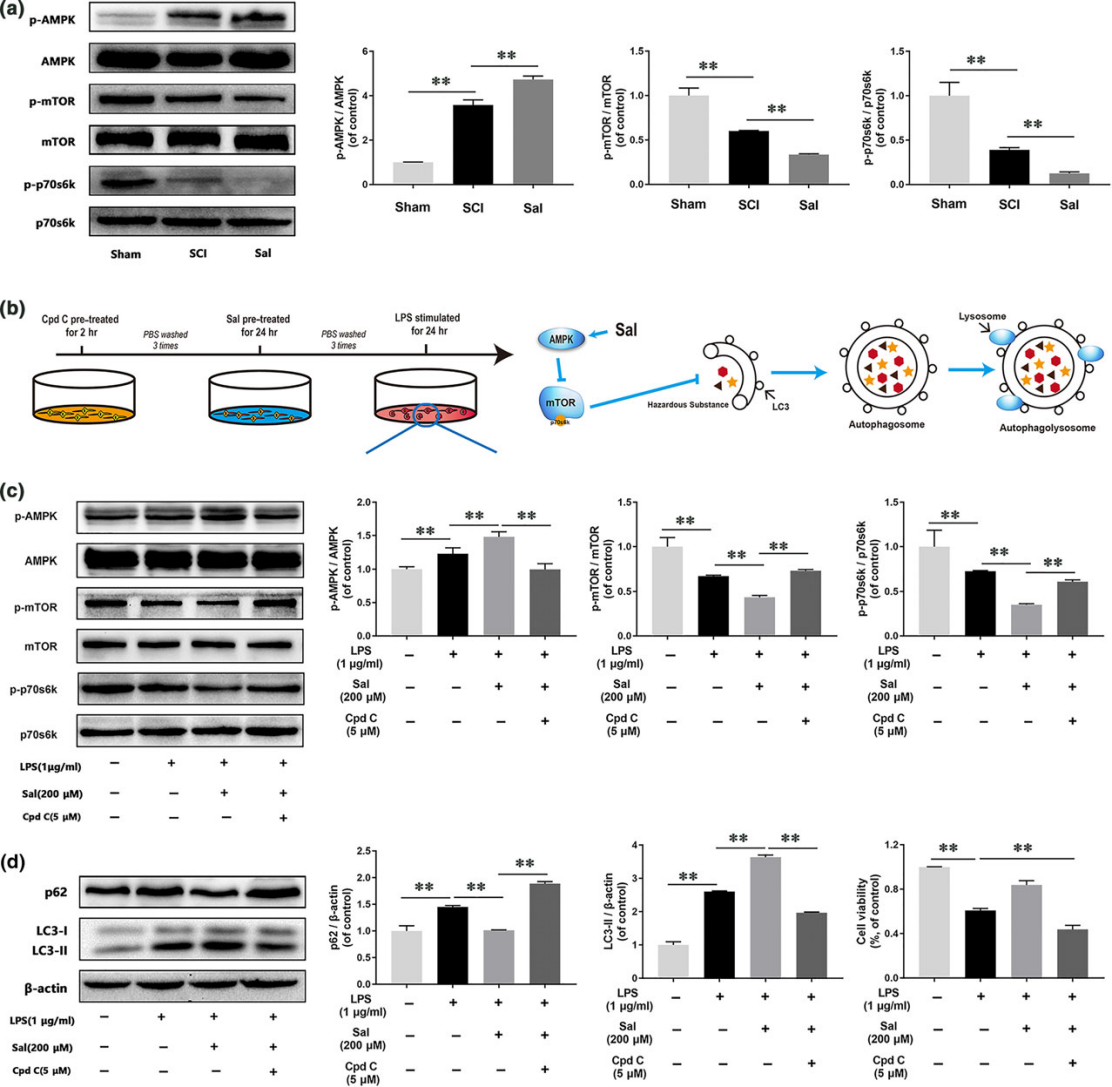
Sal promotes autophagic flux by modulation of AMPK/mTOR pathway *in vivo* and *in vitro* (**A**) Representative Western blots of and quantitative data for p‐AMPK, AMPK, p‐mTOR, mTOR, p‐p70s6k, p70s6k and β‐actin expression in each group rats. The AMPK/mTOR pathway was activated by Sal treatment in the indicated group. (**B**) Schematic of BV‐2 cell treatments and the relationship between the AMPK/mTOR pathway and autophagic flux. BV‐2 cells were pre‐treated with Cpd C for 2 hrs, followed by washing three times and treatment with Sal for 24 hrs, and then stimulated by LPS for 24 hrs. (**C, D**) Representative Western blots of and quantitative data for p‐AMPK, AMPK, p‐mTOR, mTOR, p‐p70s6k, p70s6k, p62, LC3 and β‐actin expression in each group of microglia. Sal activated autophagic flux *via* the AMPK/mTOR pathway, a change that was reversed by compound C. Densitometric analysis of all Western blot bands, whose densities were normalized to those of the corresponding total proteins or β‐actin. Data are presented as the mean ± S.D., *n *=* *3 independent experiments. Significant differences between groups are indicated as *P <* *0.05 and **P <* *0.01.

In our vivo study, we detected AMPK/mTOR pathway activity by Western blotting. As shown in Figure 8A, the expression levels of p‐AMPK were significantly increased, and the expression levels of p‐mTOR and its downstream target, p‐p70s6k, were significantly decreased in the SCI group compared with the control group. After Sal treatment, AMPK phosphorylation was increased, and mTOR and p70s6k phosphorylation was decreased in the Sal‐treated group compared with SCI group. To elucidate the mechanism by which Sal exerts its effects, our group explored whether Sal‐induced autophagic flux is associated with the AMPK/mTOR pathway *in vitro*. Microglia were treated with compound C, an AMPK inhibitor, as shown in Figure 8B. Western blot analysis of p‐AMPK, p‐mTOR and p‐p70s6k expression levels showed that Sal pre‐treatment significantly enhanced AMPK phosphorylation and reduced mTOR and p70s6k phosphorylation in the indicated group compared with the LPS‐stimulated group (Fig. 8C), implying that M1 microglia polarization may occur *via* the AMPK/mTOR pathway. Moreover, the effects of Sal were inhibited by compound C. We also detected the expression levels of the autophagic flux markers p62 and LC3‐II after the microglia were subjected to compound C pre‐treatment (Fig. 8D). The results showed that compound C inhibited Sal‐induced autophagic flux. In addition, our group also investigated the effects of Sal on the AMPK/mTOR pathway and autophagic flux, the results of which showed that Sal activated the AMPK/mTOR pathway (Fig. S4A and B). Collectively, these findings indicate that Sal acts through the AMPK/mTOR pathway to activate autophagic flux, which participates in microglia polarization.

## Discussion

SCI is a crippling and severely disabling disease characterized significant sensory and motor functional loss. Approximately 12,000 new cases occur annually 40. Numerous studies have focused on compounds that inhibit neuronal apoptosis in functional and pathological recovery after SCI 41, 42, 43, 44. In this study, we evaluated a TCM (traditional Chinese medicine) known as Sal, a compound that has shown therapeutic efficacy in SCI, and elucidated the mechanism underlying its neuroprotective effects. The results of the BBB and inclined plane tests showed that Sal treatment significantly improved motor functional recovery after injury. Moreover, H&E and Nissl staining showed that Sal prevented motor neuron loss in the ventral horn. Furthermore, more NF‐200‐positive fibres were detected at the lesion site after Sal treatment. Additionally, the expression levels of the mitochondria apoptosis‐related proteins cleaved caspase 3 and Bax were increased, while those of the mitochondria anti‐apoptosis proteins Bcl‐2 were decreased in the SCI group compared with the control group, phenomena that were reversed by Sal treatment, indicating that Sal attenuates apoptosis mediated by mitochondrial dysfunction. We also performed TUNEL staining, which measures apoptosis. The results showed that the number of TUNEL‐positive cells in the SCI group was significantly increased compared with that in the control group, an effect that was reversed by Sal treatment. These data suggested that Sal improves functional recovery after acute SCI by preventing neuron apoptosis.

After CNS injury, inflammation causes secondary damage 45 and thus exacerbates post‐SCI tissue damage and functional loss. These effects are partially attributable to the release of numerous mediators that exert cytotoxic effects on CNS cells that can lead to demyelination and neuronal loss from microglia and macrophages 46, 47, 48. However, the results of a recent investigation suggest that reductions in inflammation and immunocyte infiltration in the injured CNS may improve neuronal regeneration 49. Moreover, increasing numbers of studies have found that different macrophage polarization processes play distinct roles in tissue recovery—as some macrophages exert neurotoxic effects, while others exert neuroprotective effects 6, 50, 51, 52, 53—and that the balance between M1 and M2 polarization may partially determine the degree of CNS functional recovery experienced by patients post‐SCI. In the CNS, resident microglia and/or infiltrating monocytes from peripheral blood serve as macrophages. After SCI, M1 macrophages predominate over M2 macrophages 6, suggesting that regulating macrophage polarization in the injured spinal cord is a potential strategy for promoting locomotor recovery. Based on these findings, we investigated whether the effects of Sal in acute SCI are linked to microglia/macrophage polarization. We found that increased numbers of M1 microglia/macrophage were present at sites of injury. Interestingly, the M2/M1 ratio was significantly increased in the Sal‐treated group compared with the SCI group, suggesting that Sal decreases M1 microglia/macrophage polarization and increases M2 microglia/macrophage polarization in acute SCI.

Thus, our study focused on microglia polarization, the resident macrophages in central nervous system, and we verified that crosstalk occurs among Sal, microglia polarization and neuron apoptosis in an *in vitro* experiment using the BV‐2 microglial cell line and the PC12 neuronal cell line. Microglia mainly participate in the inflammatory response after spinal cord injury 5 and are necessary for normal neuronal function. M1 microglia are a prominent source of pro‐inflammatory factors and oxidative stress mediators, such as TNF‐α, NO and IL‐1β, which are neurotoxic 6. The pathological processes of various neurodegenerative diseases are associated with different pro‐inflammatory cytokines and chemokines whose release is elicited by M1 microglial polarization 6, thus inhibiting pro‐inflammatory mediator release by suppressing M1 polarization may have neuroprotective effects 54, 55. In the present *in vitro* study, we found that M1 microglia displayed increases in iNOS, COX‐2 mRNA and protein expression and intense CD68 staining. However, treatment with Sal prior to LPS stimulation reversed M1 polarization. Moreover, M1 microglia cocultured with neurons caused the up‐regulation of apoptosis‐related proteins, such as cleaved caspase 3 and Bax, and the down‐regulation of protective proteins, such as Bcl‐2, as well as decreases in MMP. In the coculture system, pre‐treating microglia with Sal significantly reduced apoptosis‐related protein expression levels and prevented decreases in MMP in neurons. Based on these *in vitro* results, we surmised that M1 microglia can trigger mitochondrial dysfunction and neuronal apoptosis and that Sal can suppress M1 microglia polarization and prevent the mitochondrial dysfunction‐induced neuronal apoptosis facilitated by M1 microglia. Interestingly, we found that the expression levels of the M2 microglia marker, Arg‐1, were increased after Sal treatment, even after LPS stimulation. This finding indicated that the neuroprotective effects of Sal may be associated with both the M1 and the M2 microglia populations. However, the molecular mechanism by which Sal induces changes in microglia polarization remains unclear.

Autophagy is a process of self‐digestion whereby the cell degrades useless proteins and organelles through the autophagy‐lysosome pathway to sustain cellular function 56, 57. Evidence from numerous recent studies shows that moderate autophagy exerts protective effects against various pathologies, including Alzheimer's disease 58, osteoarthritis 59 and SCI 60. Under most circumstances, autophagy has cytoprotective effects 42, 61, 62, 63; however, excessive autophagy leads to cell death. Until recently, the function of the autophagy pathway in SCI was unclear. Several recent studies have focused on the usefulness of autophagy as a therapy for inflammation‐induced disease 64, 65. Increases in the expression levels of autophagic markers have been detected following ischaemia/reperfusion or inflammation injury, indicating that injury or stress can trigger cell self‐protective mechanisms by activating autophagy 66, 67. In contrast, other studies have shown that autophagy has pernicious effects 68, 69. This discrepancy may be attributable to a lack of understanding regarding autophagic flux, which begins with formation of the autophagosome and ends with substrate degradation by the lysosome 61, 62. Interestingly, we found that the abovementioned increases in the levels of upstream autophagic markers are partially attributable lysosome dysfunction, which may suppress the cargo degradation pathway. It is well known that LC3 and p62 are symbols of autophagy and represent different autophagic processes. Specifically, LC3 represents autophagic flux initiation, while p62 represents autophagic flux termination, which is associated with lysosomal degradation 70, 71. Therefore, changes in LC3 expression are not fully indicative of autophagy‐lysosome pathway activity but are associated with changes in p62 expression. Our *in vivo* results showed that LC3 and p62 expression levels were enhanced after SCI, indicating that autophagic flux had been activated; however, the downstream processes associated with autophagic flux may have been blocked. Moreover, we found that the levels of LAMP‐2, which is the main lysosomal membrane protein and is closely associated with autophagic degradation 72, decreased significantly in the SCI group compared with the control group. These data suggest that the autophagy‐lysosome pathway is damaged in acute SCI.

**Figure 9:**
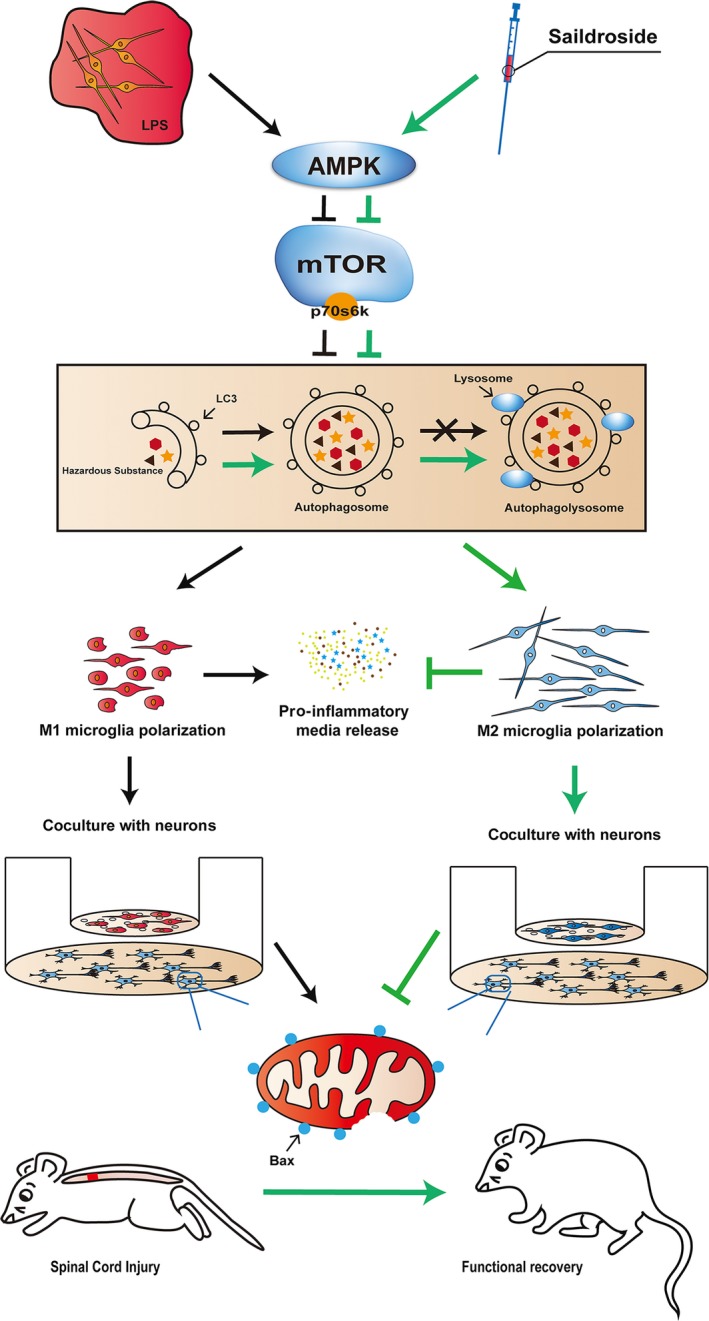
Schematic of the preventative effects of Sal on neuronal apoptosis. After LPS stimulation, resting microglia underwent M1 polarization and released pro‐inflammatory mediators to induce neuronal apoptosis. Sal enhanced the M2/M1 cell ratio by activating autophagic flux and the AMPK/mTOR pathway.

Autophagic marker expression levels are enhanced in acute inflammation; however, autophagic flux marker levels are not increased, possibly as a result of lysosomal dysfunction 70. Moreover, impaired macrophage autophagy may promote pro‐inflammatory macrophage polarization 36. The TSC1/2 complex directly suppresses the M1 macrophage response, and mTOR pathway inhibition plays an essential role in M2 activation 73. In our study, we investigated the crosstalk between microglia polarization and autophagic flux using the classic autophagy inhibitor CQ. We found that LAMP‐2 levels were decreased after LPS treatment, indicating that the autophagic degradation pathway was inhibited and that the autophagy‐lysosome pathway was disrupted. Furthermore, CQ reversed the suppressive effects of Sal on M1 microglia polarization, indicating that Sal suppressed M1 macrophage polarization through the autophagy‐lysosome pathway (Fig. 9). Consistent with our immunofluorescence results, the results of this experiment showed that M1 microglia enhance LC3 puncta and induce p62 accumulation.

Emerging reports have shown that AMPK plays a key role in cell survival and can be activated by inflammation, after which it phosphorylates the mTOR receptor to induce autophagy 74, 75. Moreover, AMPK has been demonstrated to be involved in numerous CNS diseases, such as bovine spongiform encephalopathy and Parkinson's disease, and exerts its effects through autophagic flux 76, 77. A recent study showed that the mTOR pathway plays a key role in regulating macrophage polarization 78 and that mTOR inhibition can reverse M1 macrophage polarization and promote M2 macrophage polarization 73. In this study, we detected the effects of Sal on the expression of p‐AMPK and its downstream targets, p‐mTOR and p‐p70s6k, in the rat spinal cord *in vivo* and *in vitro*. In the *in vivo* study, we demonstrated that Sal activated the AMPK/mTOR signalling pathway to promote autophagic activity. In our *in vitro* study, we found that compound C‐mediated inhibition of AMPK phosphorylation 79, 80 abrogated the effects of Sal. These findings indicated that the upstream factor of the mTOR receptor, AMPK, may be an essential target in inflammation‐induced apoptosis and may mediate the protective effects of Sal in microglia (Fig. 9). However, how Sal activates autophagic flux through the AMPK pathway remains unclear, and the relationship among inflammation, apoptosis and autophagy still needs further investigation.

## Conclusions

In summary, our study has provided evidence of the effects of Sal treatment on motor functional recovery, neuronal apoptosis, microglia polarization and autophagic flux after SCI. Our results demonstrate that Sal may be useful as a therapeutic agent that can regulate microglia polarization through the autophagy‐lysosome pathway to attenuate acute SCI.

## Conflict of Interest Statement

The authors declare no conflict of interest.

## Supporting information


**Figure S1** Sal decreases M1 microglia/macrophage in injured spinal cord. Immunofluorescence staining for CD68 in sections of injured spinal cord tissue from each group rats (scale bar: 100 μm). Sal significantly reduced the numbers of M1 microglia after SCI.
**Figure S2** Sal prevents M1 microglia‐induced neuron death. Live/dead staining results for neurons cocultured with each type of microglia (scale bar: 200 μm). Cell survival was significantly up‐regulated in cells cocultured with Sal‐pre‐treated microglia.
**Figure S3** Sal restores autophagic flux *in vivo*. (A, B, C, D) Representative western blots of and quantitative data for LAMP‐2, p62, LC3 and β‐actin expression in each group of rats. Autophagic flux was blocked, and lysosomal dysfunction occurred after SCI, while autophagic flux was restored by treatment with Sal. Densitometric analysis of all western blot bands, whose densities were normalized to those of β‐actin. Data are presented as the mean ± S.D., *s *=* *3 independent experiments. Significant differences between groups are indicated as *P <* *0.05 and **P <* *0.01.
**Figure S4.** Sal activates autophagic flux *via* the AMPK/mTOR pathway. (A, B) Representative western blots of and quantitative data for p‐AMPK, AMPK, p‐mTOR, mTOR, p‐p70s6k, p70s6k, p62, LC3 and β‐actin expression in each group of microglia. Sal activated autophagic flux *via* the AMPK/mTOR pathway. Densitometric analysis of all western blot bands, whose intensities were normalized to those of the corresponding total proteins or β‐actin. Data are presented as the mean ± S.D., *n *=* *3 independent experiments. Significant differences between groups are indicated as *P <* *0.05 and **P <* *0.01.

## References

[1] McDonald JW , Sadowsky C . Spinal‐cord injury. Lancet. 2002; 359: 417–25. 11844532 10.1016/S0140-6736(02)07603-1

[2] Tator CH , Fehlings MG . Review of the secondary injury theory of acute spinal cord trauma with emphasis on vascular mechanisms. J Neurosurg. 1991; 75: 15–26. 2045903 10.3171/jns.1991.75.1.0015

[3] Hagg T , Oudega M . Degenerative and spontaneous regenerative processes after spinal cord injury. J Neurotrauma. 2006; 23: 263–80. 10.1089/neu.2006.23.263 16629615

[4] Hall ED . Antioxidant therapies for acute spinal cord injury. Neurotherapeutics. 2011; 8: 152–67. 21424941 10.1007/s13311-011-0026-4 PMC3101837

[5] David S , Kroner A . Repertoire of microglial and macrophage responses after spinal cord injury. Nat Rev Neurosci. 2011; 12: 388–99. 21673720 10.1038/nrn3053

[6] Kigerl KA , Gensel JC , Ankeny DP , *et al* Identification of two distinct macrophage subsets with divergent effects causing either neurotoxicity or regeneration in the injured mouse spinal cord. J Neurosci. 2009; 29: 13435–44. 19864556 10.1523/JNEUROSCI.3257-09.2009 PMC2788152

[7] Shechter R , Miller O , Yovel G , *et al* Recruitment of beneficial M2 macrophages to injured spinal cord is orchestrated by remote brain choroid plexus. Immunity. 2013; 38: 555–69. 23477737 10.1016/j.immuni.2013.02.012 PMC4115271

[8] Block ML , Zecca L , Hong J‐S . Microglia‐mediated neurotoxicity: uncovering the molecular mechanisms. Nat Rev Neurosci. 2007; 8: 57–69. 17180163 10.1038/nrn2038

[9] Kroner A , Greenhalgh AD , Zarruk JG , *et al* TNF and increased intracellular iron alter macrophage polarization to a detrimental M1 phenotype in the injured spinal cord. Neuron. 2014; 83: 1098–116. 25132469 10.1016/j.neuron.2014.07.027

[10] Xu F , Huang J , He Z , *et al* Microglial polarization dynamics in dorsal spinal cord in the early stages following chronic sciatic nerve damage. Neurosci Lett. 2016; 617: 6–13. 26820376 10.1016/j.neulet.2016.01.038

[11] Cameron B , Landreth GE . Inflammation, microglia, and Alzheimer's disease. Neurobiol Dis. 2010; 37: 503–9. 19833208 10.1016/j.nbd.2009.10.006 PMC2823849

[12] Goerdt S , Orfanos CE . Other functions, other genes: alternative activation of antigen‐presenting cells. Immunity. 1999; 10: 137–42. 10072066 10.1016/s1074-7613(00)80014-x

[13] Mikita J , Dubourdieu‐Cassagno N , Deloire MS , *et al* Altered M1/M2 activation patterns of monocytes in severe relapsing experimental rat model of multiple sclerosis. Amelioration of clinical status by M2 activated monocyte administration. Mult Scler J. 2011; 17: 2–15. 10.1177/1352458510379243 20813772

[14] Bouhy D , Malgrange B , Multon S , *et al* Delayed GM‐CSF treatment stimulates axonal regeneration and functional recovery in paraplegic rats *via* an increased BDNF expression by endogenous macrophages. FASEB J. 2006; 20: 1239–41. 16636109 10.1096/fj.05-4382fje

[15] Ha Y , Kim YS , Cho JM , *et al* Role of granulocyte‐macrophage colony‐stimulating factor in preventing apoptosis and improving functional outcome in experimental spinal cord contusion injury. J Neurosurg Spine. 2005; 2: 55–61. 15658127 10.3171/spi.2005.2.1.0055

[16] Francos‐Quijorna I , Amo‐Aparicio J , Martinez‐Muriana A , *et al* IL‐4 drives microglia and macrophages toward a phenotype conducive for tissue repair and functional recovery after spinal cord injury. Glia. 2016; 64: 2079–92. 27470986 10.1002/glia.23041

[17] Ma S‐F , Chen Y‐J , Zhang J‐X , *et al* Adoptive transfer of M2 macrophages promotes locomotor recovery in adult rats after spinal cord injury. Brain Behav Immun. 2015; 45: 157–70. 25476600 10.1016/j.bbi.2014.11.007

[18] Zhang B , Bailey WM , Kopper TJ , *et al* Azithromycin drives alternative macrophage activation and improves recovery and tissue sparing in contusion spinal cord injury. J Neuroinflammation. 2015; 12: 218–230. 26597676 10.1186/s12974-015-0440-3 PMC4657208

[19] Yang X , Xu S , Qian Y , *et al* Resveratrol regulates microglia M1/M2 polarization *via* PGC‐1α in conditions of neuroinflammatory injury. Brain Behav Immun. 2017; 64: 162–172. 28268115 10.1016/j.bbi.2017.03.003

[20] Hong Z‐Y , Shi X‐R , Zhu K , *et al* SCM‐198 inhibits microglial overactivation and attenuates Aβ 1‐40‐induced cognitive impairments in rats *via* JNK and NF‐кB pathways. J Neuroinflammation. 2014; 11: 147–181. 10.1186/s12974-014-0147-x PMC4156960 25134526

[21] Rafii M , Walsh S , Little JT , *et al* A phase II trial of huperzine A in mild to moderate Alzheimer disease. Neurology. 2011; 76: 1389–94. 21502597 10.1212/WNL.0b013e318216eb7b PMC3269774

[22] Fan Z‐K , Lv G , Wang Y‐F , *et al* The protective effect of salvianolic acid B on blood–spinal cord barrier after compression spinal cord injury in rats. J Mol Neurosci. 2013; 51: 986–93. 23943397 10.1007/s12031-013-0083-8

[23] Shevtsov V , Zholus B , Shervarly V , *et al* A randomized trial of two different doses of a SHR‐5 Rhodiola rosea extract *versus* placebo and control of capacity for mental work. Phytomedicine. 2003; 10: 95–105. 12725561 10.1078/094471103321659780

[24] De Bock K , Eijnde BO , Ramaekers M , *et al* Acute Rhodiola rosea intake can improve endurance exercise performance. Int J Sport Nutr Exerc Metab. 2004; 14: 298–307. 15256690 10.1123/ijsnem.14.3.298

[25] Maslov L , Lishmanov YB , Arbuzov A , *et al* Antiarrhythmic activity of phytoadaptogens in short‐term ischemia‐reperfusion of the heart and postinfarction cardiosclerosis. Bull Exp Biol Med. 2009; 147: 331–4. 19529855 10.1007/s10517-009-0502-6

[26] Qu Z‐Q , Zhou Y , Zeng Y‐S , Lin Y‐K , Li Y , Zhong Z‐Q , Chan WY . Protective effects of a Rhodiola crenulata extract and salidroside on hippocampal neurogenesis against streptozotocin‐induced neural injury in the rat. PloS one. 2012; 7: e29641. 22235318 10.1371/journal.pone.0029641 PMC3250459

[27] Sun L , Isaak CK , Zhou Y , *et al* Salidroside and tyrosol from Rhodiola protect H9c2 cells from ischemia/reperfusion‐induced apoptosis. Life Sci. 2012; 91: 151–8. 22771701 10.1016/j.lfs.2012.06.026

[28] Song B , Huang G , Xiong Y , *et al* Inhibitory effects of salidroside on nitric oxide and prostaglandin E2 production in lipopolysaccharide‐stimulated RAW 264.7 macrophages. J Med Food. 2013; 16: 997–1003. 24180550 10.1089/jmf.2012.2473

[29] Rubinsztein DC , Mariño G , Kroemer G . Autophagy and aging. Cell. 2011; 146: 682–95. 21884931 10.1016/j.cell.2011.07.030

[30] Kraft C , Martens S . Mechanisms and regulation of autophagosome formation. Curr Opin Cell Biol. 2012; 24: 496–501. 22664348 10.1016/j.ceb.2012.05.001

[31] Mizushima N , Levine B , Cuervo AM , *et al* Autophagy fights disease through cellular self‐digestion. Nature. 2008; 451: 1069–75. 18305538 10.1038/nature06639 PMC2670399

[32] Levine B , Kroemer G . Autophagy in the pathogenesis of disease. Cell. 2008; 132: 27–42. 18191218 10.1016/j.cell.2007.12.018 PMC2696814

[33] Goldshmit Y , Kanner S , Zacs M , *et al* Rapamycin increases neuronal survival, reduces inflammation and astrocyte proliferation after spinal cord injury. Mol Cell Neurosci. 2015; 68: 82–91. 25936601 10.1016/j.mcn.2015.04.006

[34] Chen P , Cescon M , Bonaldo P . Autophagy‐mediated regulation of macrophages and its applications for cancer. Autophagy. 2014; 10: 192–200. 24300480 10.4161/auto.26927 PMC5396097

[35] Yang M , Liu J , Shao J , *et al* Cathepsin S‐mediated autophagic flux in tumor‐associated macrophages accelerate tumor development by promoting M2 polarization. Mol Cancer. 2014; 13: 43–57. 24580730 10.1186/1476-4598-13-43 PMC4015740

[36] Liu K , Zhao E , Ilyas G , *et al* Impaired macrophage autophagy increases the immune response in obese mice by promoting proinflammatory macrophage polarization. Autophagy. 2015; 11: 271–84. 25650776 10.1080/15548627.2015.1009787 PMC4502775

[37] Basso DM , Beattie MS , Bresnahan JC . A sensitive and reliable locomotor rating scale for open field testing in rats. J Neurotrauma. 1995; 12: 1–21. 7783230 10.1089/neu.1995.12.1

[38] Perrin FE , Boniface G , Serguera C , *et al* Grafted human embryonic progenitors expressing neurogenin‐2 stimulate axonal sprouting and improve motor recovery after severe spinal cord injury. PLoS ONE. 2010; 5: e15914–e15920. 21209909 10.1371/journal.pone.0015914 PMC3012721

[39] Blasi E , Barluzzi R , Bocchini V , *et al* Immortalization of murine microglial cells by a v‐raf/v‐myc carrying retrovirus. J Neuroimmunol. 1990; 27: 229–37. 2110186 10.1016/0165-5728(90)90073-v

[40] Saunders LL , Clarke A , Tate DG , *et al* Lifetime prevalence of chronic health conditions among persons with spinal cord injury. Arch Phys Med Rehabil. 2015; 96: 673–9. 25497516 10.1016/j.apmr.2014.11.019

[41] Cavallucci V , D'Amelio M . Matter of life and death: the pharmacological approaches targeting apoptosis in brain diseases. Curr Pharm Des. 2011; 17: 215–29. 21348825 10.2174/138161211795049705

[42] Li H‐T , Zhao X‐Z , Zhang X‐R , *et al* Exendin‐4 enhances motor function recovery *via* promotion of autophagy and inhibition of neuronal apoptosis after spinal cord injury in rats. Mol Neurobiol. 2016; 53: 4073–82. 26198566 10.1007/s12035-015-9327-7

[43] Tang P , Hou H , Zhang L , *et al* Autophagy reduces neuronal damage and promotes locomotor recovery *via* inhibition of apoptosis after spinal cord injury in rats. Mol Neurobiol. 2014; 49: 276–87. 23954967 10.1007/s12035-013-8518-3

[44] Zhang J , Cui Z , Feng G , *et al* RBM5 and p53 expression after rat spinal cord injury: implications for neuronal apoptosis. Int J Biochem Cell Biol. 2015; 60: 43–52. 25578565 10.1016/j.biocel.2014.12.020

[45] Popovich P , Stokes B , Whitacre C . Concept of autoimmunity following spinal cord injury: possible roles for T lymphocytes in the traumatized central nervous system. J Neurosci Res. 1996; 45: 349–63. 8872895 10.1002/(SICI)1097-4547(19960815)45:4<349::AID-JNR4>3.0.CO;2-9

[46] David S , Lopez‐Vales R , Wee YV . Harmful and beneficial effects of inflammation after spinal cord injury: potential therapeutic implications. Handb Clin Neurol. 2011; 109: 485–502. 10.1016/B978-0-444-52137-8.00030-9 23098732

[47] Hawthorne AL , Popovich PG . Emerging concepts in myeloid cell biology after spinal cord injury. Neurotherapeutics. 2011; 8: 252–61. 21400005 10.1007/s13311-011-0032-6 PMC3101835

[48] Ren Y , Young W . Managing inflammation after spinal cord injury through manipulation of macrophage function. Neural Plast. 2013; 2013: 945034–945042. 24288627 10.1155/2013/945034 PMC3833318

[49] Bowes AL , Yip PK . Modulating inflammatory cell responses to spinal cord injury: all in good time. J Neurotrauma. 2014; 31: 1753–66. 24934600 10.1089/neu.2014.3429

[50] Ishii H , Jin X , Ueno M , *et al* Adoptive transfer of Th1‐conditioned lymphocytes promotes axonal remodeling and functional recovery after spinal cord injury. Cell Death Dis. 2012; 3: e363–e372. 22875000 10.1038/cddis.2012.106 PMC3434665

[51] Kipnis J , Mizrahi T , Hauben E , *et al* Neuroprotective autoimmunity: naturally occurring CD4 + CD25 + regulatory T cells suppress the ability to withstand injury to the central nervous system. Proc Natl Acad Sci. 2002; 99: 15620–5. 12429857 10.1073/pnas.232565399 PMC137766

[52] Plemel JR , Yong VW , Stirling DP . Immune modulatory therapies for spinal cord injury–past, present and future. Exp Neurol. 2014; 258: 91–104. 25017890 10.1016/j.expneurol.2014.01.025

[53] Thawer SG , Mawhinney L , Chadwick K , *et al* Temporal changes in monocyte and macrophage subsets and microglial macrophages following spinal cord injury in the Lys‐Egfp‐ki mouse model. J Neuroimmunol. 2013; 261: 7–20. 23711349 10.1016/j.jneuroim.2013.04.008

[54] Yrjänheikki J , Keinänen R , Pellikka M , *et al* Tetracyclines inhibit microglial activation and are neuroprotective in global brain ischemia. Proc Natl Acad Sci. 1998; 95: 15769–74. 9861045 10.1073/pnas.95.26.15769 PMC28119

[55] Chen Y , Won S , Xu Y , *et al* Targeting microglial activation in stroke therapy: pharmacological tools and gender effects. Curr Med Chem. 2014; 21: 2146–55. 24372213 10.2174/0929867321666131228203906 PMC4076056

[56] Dutta D , Xu J , Kim J‐S , *et al* Upregulated autophagy protects cardiomyocytes from oxidative stress‐induced toxicity. Autophagy. 2013; 9: 328–44. 23298947 10.4161/auto.22971 PMC3590254

[57] Yang Z , Klionsky DJ . Eaten alive: a history of macroautophagy. Nat Cell Biol. 2010; 12: 814–22. 20811353 10.1038/ncb0910-814 PMC3616322

[58] Gu P , Jakkoju A , Wang M , *et al* Autophagy and its neuroprotection in neurodegenerative diseases. Neural Regener Res. 2011; 6: 1765–74.

[59] Chang J , Wang W , Zhang H , *et al* The dual role of autophagy in chondrocyte responses in the pathogenesis of articular cartilage degeneration in osteoarthritis. Int J Mol Med. 2013; 32: 1311–8. 24126970 10.3892/ijmm.2013.1520

[60] Zhou K‐L , Zhou Y‐F , Wu K , *et al* Stimulation of autophagy promotes functional recovery in diabetic rats with spinal cord injury. Sci Rep. 2015; 5: 1713–1727. 10.1038/srep17130 PMC4657088 26597839

[61] Levine B , Klionsky DJ . Development by self‐digestion: molecular mechanisms and biological functions of autophagy. Dev Cell. 2004; 6: 463–77. 15068787 10.1016/s1534-5807(04)00099-1

[62] Mizushima N . Autophagy in protein and organelle turnover. Cold Spring Harbor symposia on quantitative biology. 2011; 76: 397–402. 21813637 10.1101/sqb.2011.76.011023

[63] Ding F , Shao Z‐W , Xiong L‐M . Cell death in intervertebral disc degeneration. Apoptosis. 2013; 18: 777–85. 23512131 10.1007/s10495-013-0839-1

[64] Ling H , Chen H , Wei M , *et al* The effect of autophagy on inflammation cytokines in renal ischemia/reperfusion injury. Inflammation. 2016; 39: 347–56. 26412257 10.1007/s10753-015-0255-5

[65] Fan X , Wang J , Hou J , *et al* Berberine alleviates ox‐LDL induced inflammatory factors by up‐regulation of autophagy *via* AMPK/mTOR signaling pathway. J Transl Med. 2015; 13: 92–102. 25884210 10.1186/s12967-015-0450-z PMC4365560

[66] Kanno H , Ozawa H , Sekiguchi A , *et al* The role of autophagy in spinal cord injury. Autophagy. 2009; 5: 390–2. 19158496 10.4161/auto.5.3.7724

[67] Kanno H , Ozawa H , Sekiguchi A , *et al* The role of mTOR signaling pathway in spinal cord injury. Cell Cycle. 2012; 11: 3175–9. 22895182 10.4161/cc.21262 PMC3466516

[68] Chen J‐W , Ni B‐B , Li B , *et al* The responses of autophagy and apoptosis to oxidative stress in nucleus pulposus cells: implications for disc degeneration. Cell Physiol Biochem. 2014; 34: 1175–89. 25277442 10.1159/000366330

[69] Tang Y , Jacobi A , Vater C , *et al* Icariin promotes angiogenic differentiation and prevents oxidative stressotes angiogenic differentiation and prevents oxi. Stem Cells. 2015; 33: 1863–77. 25787271 10.1002/stem.2005

[70] Chen S , Yuan J , Yao S , *et al* Lipopolysaccharides may aggravate apoptosis through accumulation of autophagosomes in alveolar macrophages of human silicosis. Autophagy. 2015; 11: 2346–57. 26553601 10.1080/15548627.2015.1109765 PMC4835201

[71] Liu S , Sarkar C , Dinizo M , *et al* Disrupted autophagy after spinal cord injury is associated with ER stress and neuronal cell death. Cell Death Dis. 2015; 6: e1582. 25569099 10.1038/cddis.2014.527 PMC4669738

[72] Eskelinen E‐L . Roles of LAMP‐1 and LAMP‐2 in lysosome biogenesis and autophagy. Mol Aspects Med. 2006; 27: 495–502. 16973206 10.1016/j.mam.2006.08.005

[73] Zhu L , Yang T , Li L , *et al* TSC1 controls macrophage polarization to prevent inflammatory disease. Nat Commun. 2014; 5: 4696–4708. 25175012 10.1038/ncomms5696

[74] Yue W , Yang CS , DiPaola RS , *et al* Repurposing of metformin and aspirin by targeting AMPK‐mTOR and inflammation for pancreatic cancer prevention and treatment. Cancer Prev Res. 2014; 7: 388–97. 10.1158/1940-6207.CAPR-13-0337 24520038

[75] Ashabi G , Khalaj L , Khodagholi F , *et al* Pre‐treatment with metformin activates Nrf2 antioxidant pathways and inhibits inflammatory responses through induction of AMPK after transient global cerebral ischemia. Metab Brain Dis. 2015; 30: 747–54. 25413451 10.1007/s11011-014-9632-2

[76] Lee J‐H , Jeong J‐K , Park S‐Y . Sulforaphane‐induced autophagy flux prevents prion protein‐mediated neurotoxicity through AMPK pathway. Neuroscience. 2014; 278: 31–9. 25130556 10.1016/j.neuroscience.2014.07.072

[77] Hou Y‐S , Guan J‐J , Xu H‐D , *et al* Sestrin2 protects dopaminergic cells against rotenone toxicity through AMPK‐dependent autophagy activation. Mol Cell Biol. 2015; 35: 2740–51. 26031332 10.1128/MCB.00285-15 PMC4508325

[78] Byles V , Covarrubias AJ , Ben‐Sahra I , *et al* The TSC‐mTOR pathway regulates macrophage polarization. Nat Commun. 2013; 4: 2834–2844. 24280772 10.1038/ncomms3834 PMC3876736

[79] Lamberts RR , Onderwater G , Hamdani N , *et al* Reactive oxygen species–induced stimulation of 5′ AMP‐activated protein kinase mediates sevoflurane‐induced cardioprotection. Circulation. 2009; 120: S10–5. 19752353 10.1161/CIRCULATIONAHA.108.828426

[80] Yang L , Zhu L , Dong W , *et al* Reactive oxygen species‐mediated mitochondrial dysfunction plays a critical role in high glucose‐induced nucleus pulposus cell injury. Int Orthop. 2014; 38: 205–206. 10.1007/s00264-013-2144-6 PMC3890110 24122049

