# Supplementary material for: Salidroside attenuates neuroinflammation and improves functional recovery after spinal cord injury through microglia polarization regulation
Source: J Cell Mol Med. 2017 Nov 17;22(2):1148–66. doi: 10.1111/jcmm.13368 (PMC5783886; doi:10.1111/jcmm.13368)
Supplement: Supplementary file 1 — Figure S1 Sal decreases M1 microglia/macrophage in injured spinal cord. Immunofluorescence staining for CD68 in sections of injured spinal cord tissue from each group rats (scale bar: 100 μm). Sal significantly reduced the numbers of M1 microglia after SCI. Figure S2 Sal prevents M1 microglia‐induced neuron death. Live/dead staining results for neurons cocultured with each type of microglia (scale bar: 200 μm). Cell survival was significantly up‐regulated in cells cocultured with Sal‐pre‐treated microglia. Figure S3 Sal restores autophagic flux in vivo. (A, B, C, D) Representative western blots of and quantitative data for LAMP‐2, p62, LC3 and β‐actin expression in each group of rats. Autophagic flux was blocked, and lysosomal dysfunction occurred after SCI, while autophagic flux was restored by treatment with Sal. Densitometric analysis of all western blot bands, whose densities were normalized to those of β‐actin. Data are presented as the mean ± S.D., s = 3 independent experiments. Significant differences between groups are indicated as *P < 0.05 and **P < 0.01. Figure S4. Sal activates autophagic flux via the AMPK/mTOR pathway. (A, B) Representative western blots of and quantitative data for p‐AMPK, AMPK, p‐mTOR, mTOR, p‐p70s6k, p70s6k, p62, LC3 and β‐actin expression in each group of microglia. Sal activated autophagic flux via the AMPK/mTOR pathway. Densitometric analysis of all western blot bands, whose intensities were normalized to those of the corresponding total proteins or β‐actin. Data are presented as the mean ± S.D., n = 3 independent experiments. Significant differences between groups are indicated as *P < 0.05 and **P < 0.01. [file JCMM-22-1148-s001.pdf]

Figure S1

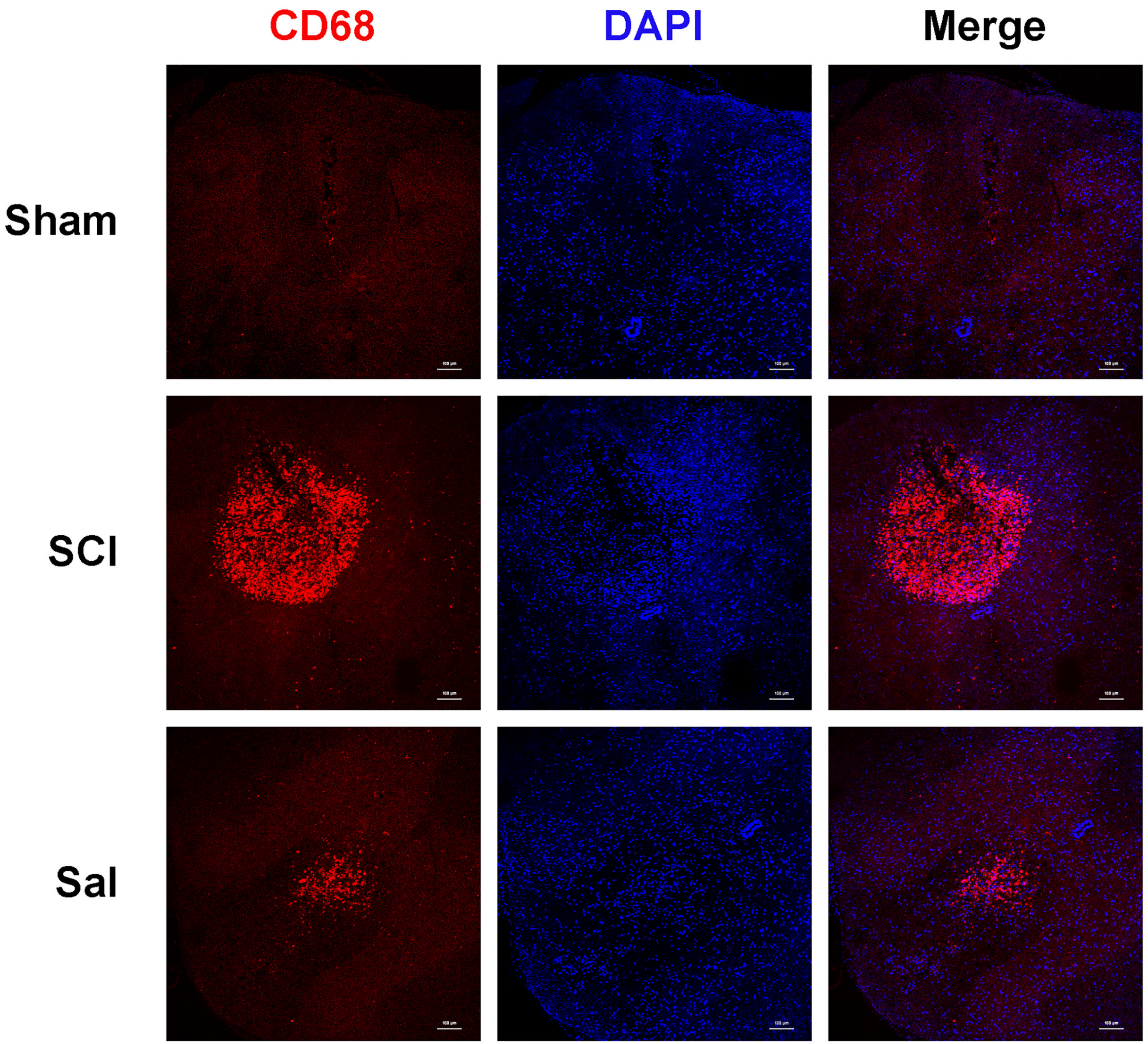

Figure S1 Sal decreases M1 microglia/macrophage in injured spinal cord. Immunofluorescence staining for CD68 in sections of injured spinal cord tissue from each group rats (scale bar: 100 μm). Sal significantly reduced the numbers of M1 microglia after SCI.

Figure S2

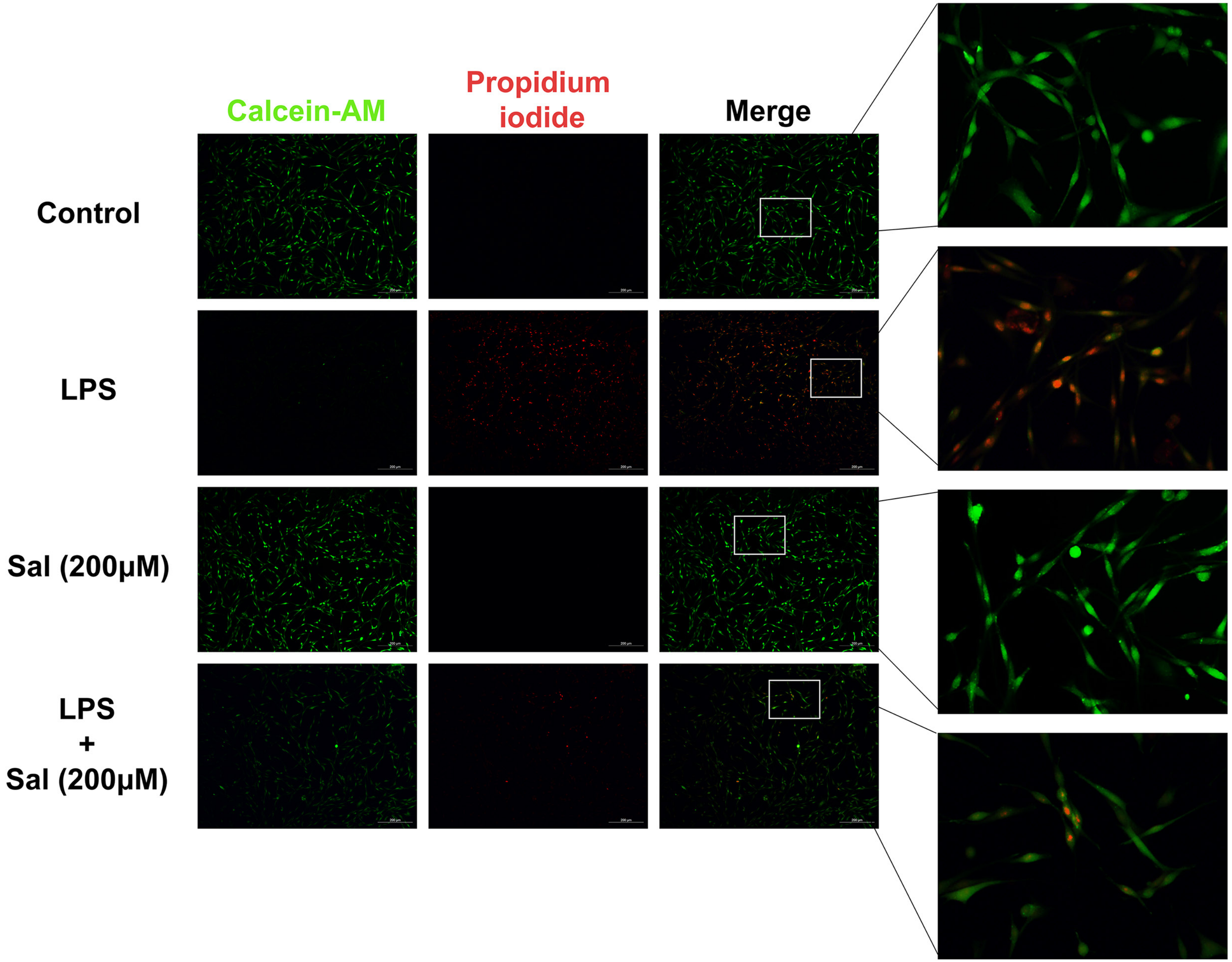

Figure S2 Sal prevents M1 microglia-induced neuron death. Live/dead staining results for neurons co-cultured with each type of microglia (scale bar: 200 μm). Cell survival was significantly upregulated in cells co-cultured with Sal-pretreated microglia.

Figure S3

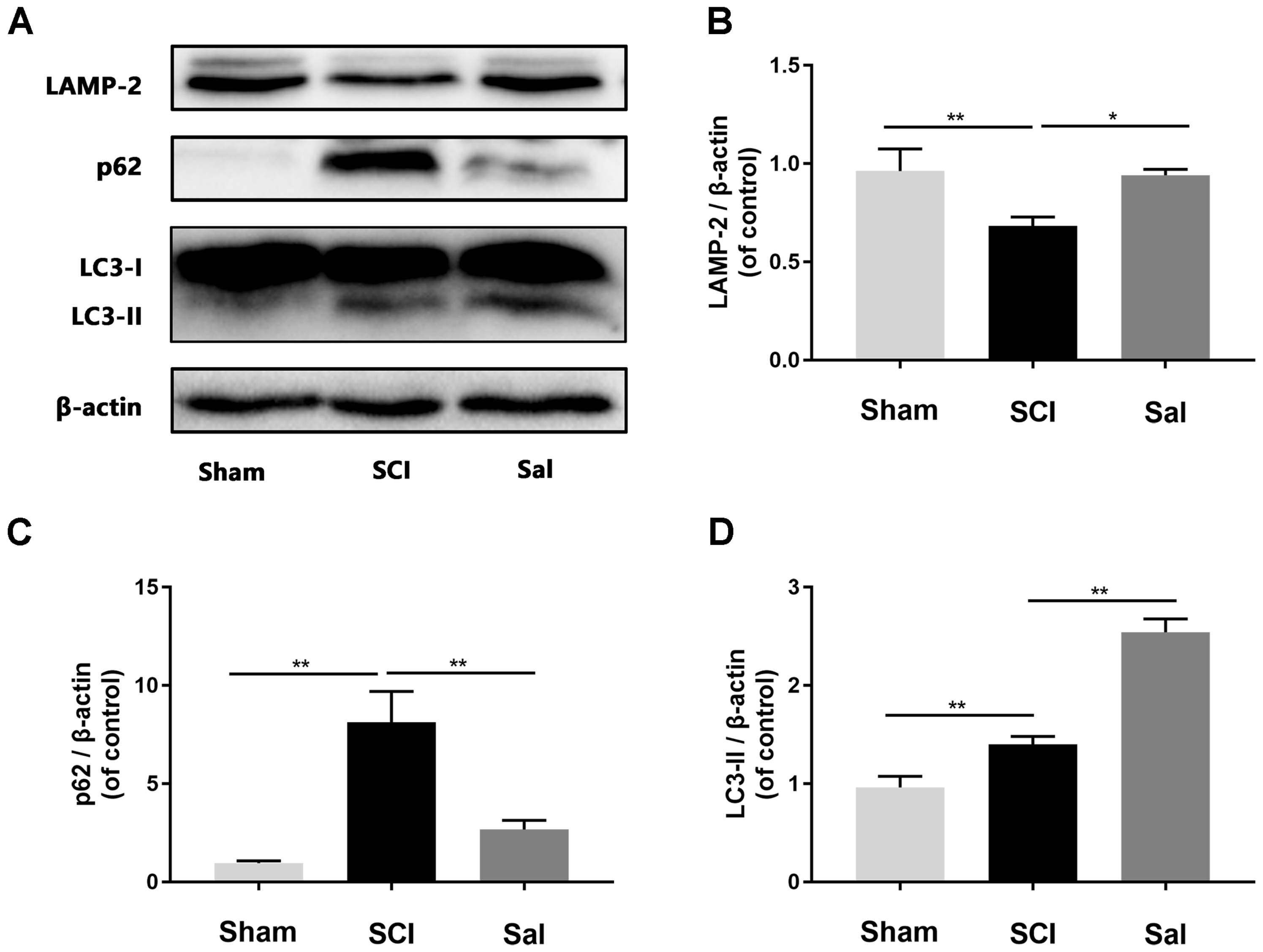

Figure S3 Sal restores autophagic flux *in vivo*. (A, B, C, D) Representative western blots of and quantitative data for LAMP-2, p62, LC3 and β-actin expression in each group of rats. Autophagic flux was blocked, and lysosomal dysfunction occurred after SCI, while autophagic flux was restored by treatment with Sal. Densitometric analysis of all western blot bands, whose densities were normalized to those of β-actin. Data are presented as the mean ± SD, n = 3 independent experiments. Significant differences between groups are indicated as $^*P < 0.05$ and $^{**}P < 0.01$.

Figure S4

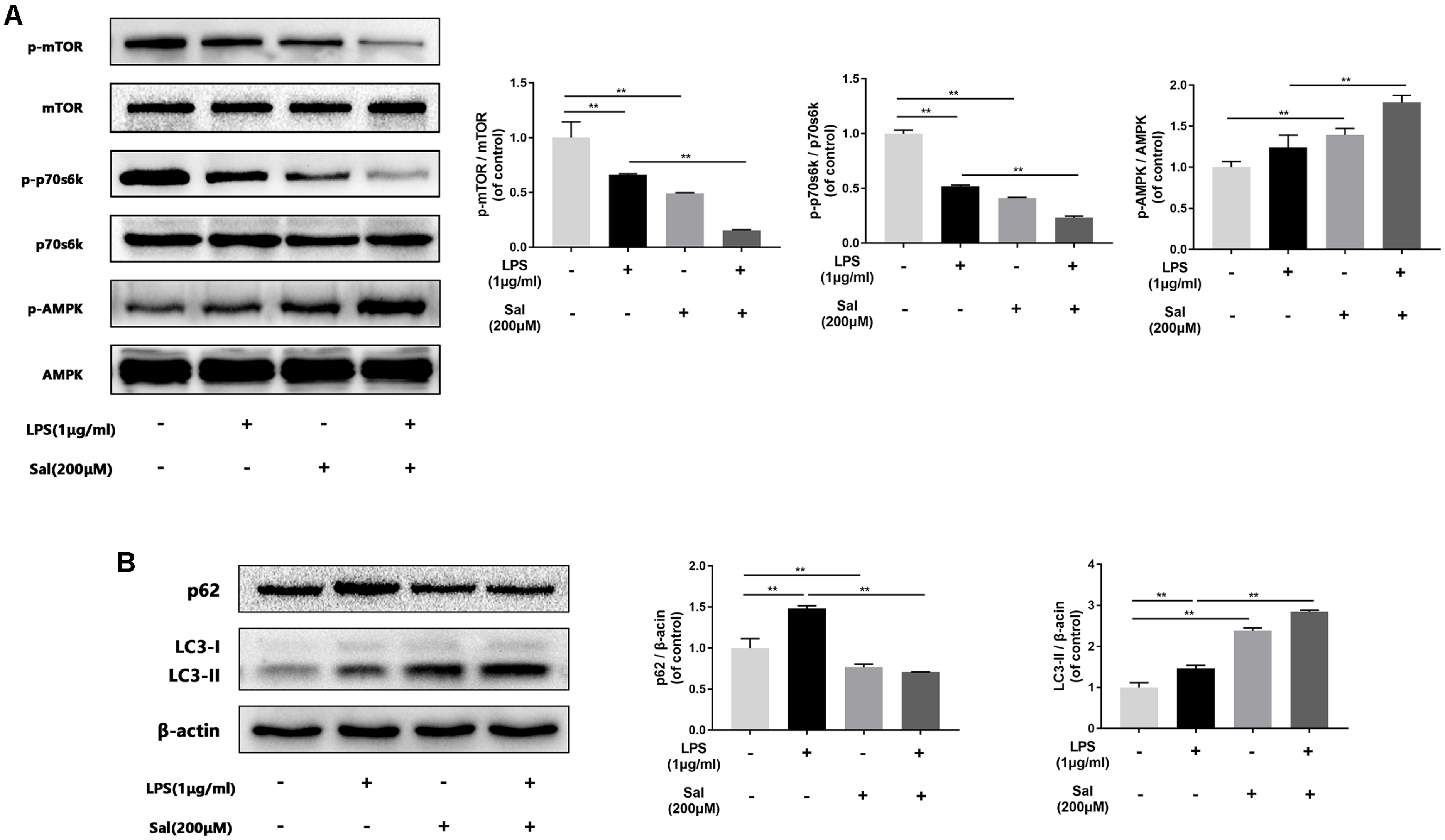

Figure S4 Sal activates autophagic flux via the AMPK/mTOR pathway. (A, B) Representative western blots of and quantitative data for pAMPK, AMPK, p-mTOR, mTOR, p-p70s6k, p70s6k, p62, LC3 and β-actin expression in each group of microglia. Sal activated autophagic flux via the AMPK/mTOR pathway. Densitometric analysis of all western blot bands, whose intensities were normalized to those of the corresponding total proteins or β-actin. Data are presented as the mean ± SD, n = 3 independent experiments. Significant differences between groups are indicated as *P < 0.05 and **P < 0.01.
